# Machine learning-based high-frequency neuronal spike reconstruction from low-frequency and low-sampling-rate recordings

**DOI:** 10.1038/s41467-024-44794-2

**Published:** 2024-01-20

**Authors:** Nari Hong, Boil Kim, Jaewon Lee, Han Kyoung Choe, Kyong Hwan Jin, Hongki Kang

**Affiliations:** 1 Department of Electrical Engineering and Computer Science, Daegu Gyeongbuk Institute of Science and Technology (DGIST), Daegu, 42988 Republic of Korea; 2 Information and Communication Engineering Research Center, Daegu Gyeongbuk Institute of Science and Technology (DGIST), Daegu, 42988 Republic of Korea; 3 Department of Brain Sciences, Daegu Gyeongbuk Institute of Science and Technology (DGIST), Daegu, 42988 Republic of Korea; 4 School of Electrical Engineering, Korea University, Seoul, 02841 Republic of Korea

**Keywords:** Biomedical engineering, Electrical and electronic engineering

## Abstract

Recording neuronal activity using multiple electrodes has been widely used to understand the functional mechanisms of the brain. Increasing the number of electrodes allows us to decode more variety of functionalities. However, handling massive amounts of multichannel electrophysiological data is still challenging due to the limited hardware resources and unavoidable thermal tissue damage. Here, we present machine learning (ML)-based reconstruction of high-frequency neuronal spikes from subsampled low-frequency band signals. Inspired by the equivalence between high-frequency restoration and super-resolution in image processing, we applied a transformer ML model to neuronal data recorded from both in vitro cultures and in vivo male mouse brains. Even with the x8 downsampled datasets, our trained model reasonably estimated high-frequency information of spiking activity, including spike timing, waveform, and network connectivity. With our ML-based data reduction applicable to existing multichannel recording hardware while achieving neuronal signals of broad bandwidths, we expect to enable more comprehensive analysis and control of brain functions.

## Abstract (web summary)

Multichannel neural recording enhances understanding of brain function, but handling large data is challenging. Here, the authors develop machine learning-based high frequency spike reconstruction from subsampled low-frequency neuronal signals.

## Introduction

Multichannel recording of neuronal activity is the key to brain-machine interfaces (BMIs), enabling the decoding of motor intentions or brain functional connectivity^1–3^. Extracellular signals such as spikes and local field potentials (LFPs) recorded by multiple implanted electrodes have been used for BMI technologies. Recent advancements in neural recording hardware have focused on increasing the number of simultaneous recording electrodes to obtain richer information for detailed network analysis^4–7^. The more electrode data we record, the wider variety of functions we can classify for the precise associated operation. Simultaneously, numerous efforts have been made to implement untethered and wireless data transfer for efficient long-term implantation of recording systems^8^. With these efforts, a wireless device has been demonstrated to show real-time operation in implanted primates^9^. Despite these advances, current BMI technologies have limitations in processing large amounts of neural data. Most BMI systems adopt single-unit spiking activity for enhanced decoding performance; however, in order to capture individual spikes that typically occur in a millisecond timescale, the recording instrument needs a sampling rate of at least 10 kHz or higher. Moreover, higher numbers of electrodes require larger storage memory and induce higher power consumption in recording and wireless communication, resulting in significant heat dissipation. Such heating should be avoided in implantable BMIs because it can cause thermal damage to surrounding biological tissues, especially for CMOS-based active neural probes that multiplexing electronics are integrated into the probe shank^5^ or for wireless neural sensors that are fully implanted^10–12^.

To ease these constraints by reducing the recording data volumes, several techniques to lower data samplings such as adaptive sampling^13,14^, compressed sensing^15^, spiking-band power-based decoding^16^, downgrading signal qualities^17^, on-chip compression^18,19^, and on-chip spike detection^20^ have been suggested. While these approaches have shown promising results in significant data reduction, their applications in advanced BMIs are still limited. Broader bandwidths of neuronal signals, not only high-frequency band spikes but also lower-frequency band signals such as LFPs, strongly correlate with brain functions that are essential for BMIs^21^. In addition, recent techniques using LFPs alone or in combination with spikes have been proposed to compensate for the major limitations of spike-based BMIs: the difficulty of consistent long-term measurement and the need for high-sampling recording^22–25^. However, the existing data reduction algorithms are designed to focus primarily on spiking activities, thereby challenging to apply to lower-frequency signals and providing limited information. Moreover, existing approaches often require custom-designed recording hardware for on-chip signal pre-processing before data transmission, such as encoders for implementing individual algorithms or additional circuitries for detecting spikes by identifying threshold-crossing events or characterizing spike waveforms and for converting spikes into binary form. This also limits universal applicability to state-of-the-art BMI technologies. Therefore, there is a strong need to develop a neural data reduction algorithm that is universally applicable without constraints on signal types and compatible with common recording hardware.

**Fig. 1:**
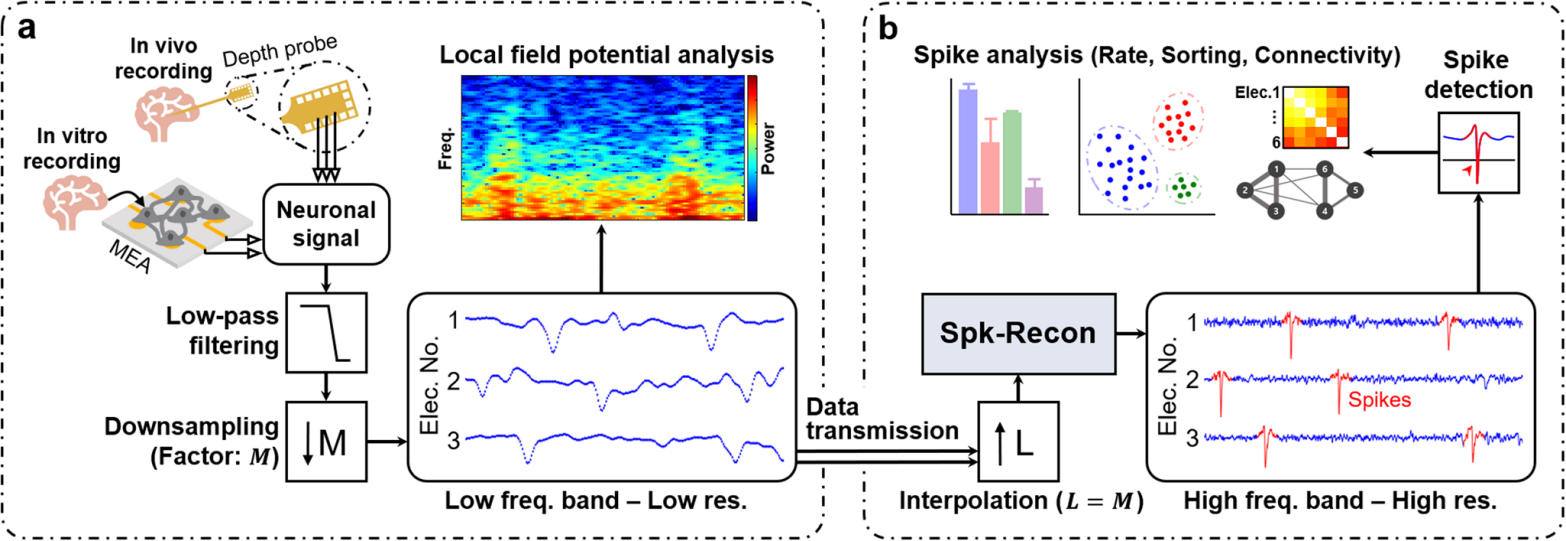
Reconstruction of high-frequency neuronal spikes from downsampled low-frequency band datasets using a machine learning model. **a** Multichannel neural recording of low-frequency (Low freq. band) signals with low temporal resolution (Low res.). The low-pass filter used is a fourth-order Butterworth filter, which is realistic. **b** Machine learning (ML)-based restoration of high-frequency (High freq. band) spikes with high temporal resolution (High res.). The pre-interpolation is performed by the Fourier method^27^. The Spk-Recon ML model is based on a Swin transformer^28,29^.

In this work, we present a machine learning (ML) framework for reconstructing high-frequency neuronal spikes from subsampled low-frequency signals (Fig. 1). Our approach can reduce neural recording data volume through low-pass filtering and simple downsampling of acquired neuronal signals. Feeding this downsampled low-pass filtered (LPF) data into an ML model combined with signal interpolation can restore high-frequency neuronal signals with high temporal resolution. It enables recording data reduction while simultaneously obtaining neuronal signals of broad bandwidths. We hypothesized that the ML models introduced for the image super-resolution task, which recovers high-quality images from low-resolution images by restoring high-frequency details^26^, can fit into our spike restoration problem. To test this hypothesis, we leveraged a transformer, one of the state-of-the-art ML models for image super-resolution, to build a spike reconstruction ML model named Spk-Recon that uses subsampled low-frequency neural recording data as an input. We applied the Spk-Recon model to multichannel neural recording datasets from in vitro hippocampal neurons and in vivo mouse brains. Through conventional quantitative spike analyses for timing and waveform, we demonstrated that our Spk-Recon model could reconstruct accurate spikes from significantly downsampled low-frequency neuronal signals, with a hit rate of spike occurrence approaching 0.8−0.9 and a clustering accuracy of spike sorting over 96%.

## Results

### ML framework for high-frequency neuronal spike reconstruction

The pipeline of our proposed approach for neuronal spike reconstruction is as follows: multichannel neural recording of low-frequency signals with low temporal resolution (Fig. 1a) and ML-based restoration of high-frequency spikes with high temporal resolution (Fig. 1b). First, neuronal data is collected under in vitro or in vivo conditions, and lower frequency band signals with the reduced resolution are obtained through low-pass filtering and sampling at a low sampling rate (Fig. 1a). These low-frequency signals contain typical frequency components of LFPs; it is possible to apply fundamental spectral analyses to the acquired datasets for characterizing brain dynamics. To realize the neural recording of low-frequency and low-resolution signals, we measured electrical signals, sampled at 25 kHz, from in vitro rat hippocampal cultures using a planar microelectrode array (MEA) and in vivo mouse brains using a penetrating depth probe. The recorded signals were passed through a fourth-order Butterworth low-pass filter (cutoff frequency of 200 Hz) and then subsampled by a predetermined downsampling factor ($M$ in Fig. 1a: 1, 8, 16, or 25). Here, we recorded neuronal signals with a wide frequency range at a high sampling rate and then filtered and subsampled them to obtain downsampled LPF inputs for the ML model. This was intended to get the corresponding high-frequency and high-sampled ground truth (GT) signals for model training and reconstruction performance assessment. The actual situation in which we apply the model would be recording low-frequency signals at a low sampling rate.

Next, the acquired low-frequency and low-resolution data is fed into an ML model, Spk-Recon, based on a transformer architecture to reconstruct high-frequency and high temporal resolution neuronal signals (Fig. 1b). The main difference from previous transformer-based works for image super-resolution is that our approach has a pre-interpolation process before putting the data into the model. This is to enhance the resolution of the downsampled LPF signals to a desired higher temporal resolution, which will be the output resolution. Lastly, high-frequency, high-resolution neuronal signals are predicted by forwarding the pre-interpolated signals to the Spk-Recon model. Detecting spikes in these output signals allows classical spike train analyses such as spike rate, sorting, and functional connectivity. To implement these processes, we first interpolated the downsampled LPF datasets via the Fourier method^27^. The interpolation factor ($L$ in Fig. 1b) was set equal to the downsampling factor ($M$ in Fig. 1a) so that the temporal resolution of the final reconstructed signals through the model was the same as that of the original high-sampled signals before the downsampling in Fig. 1a. The restored outputs were compared with high-frequency GT spikes measured at the high sampling rate to evaluate the performance of our proposed framework.

**Fig. 2:**
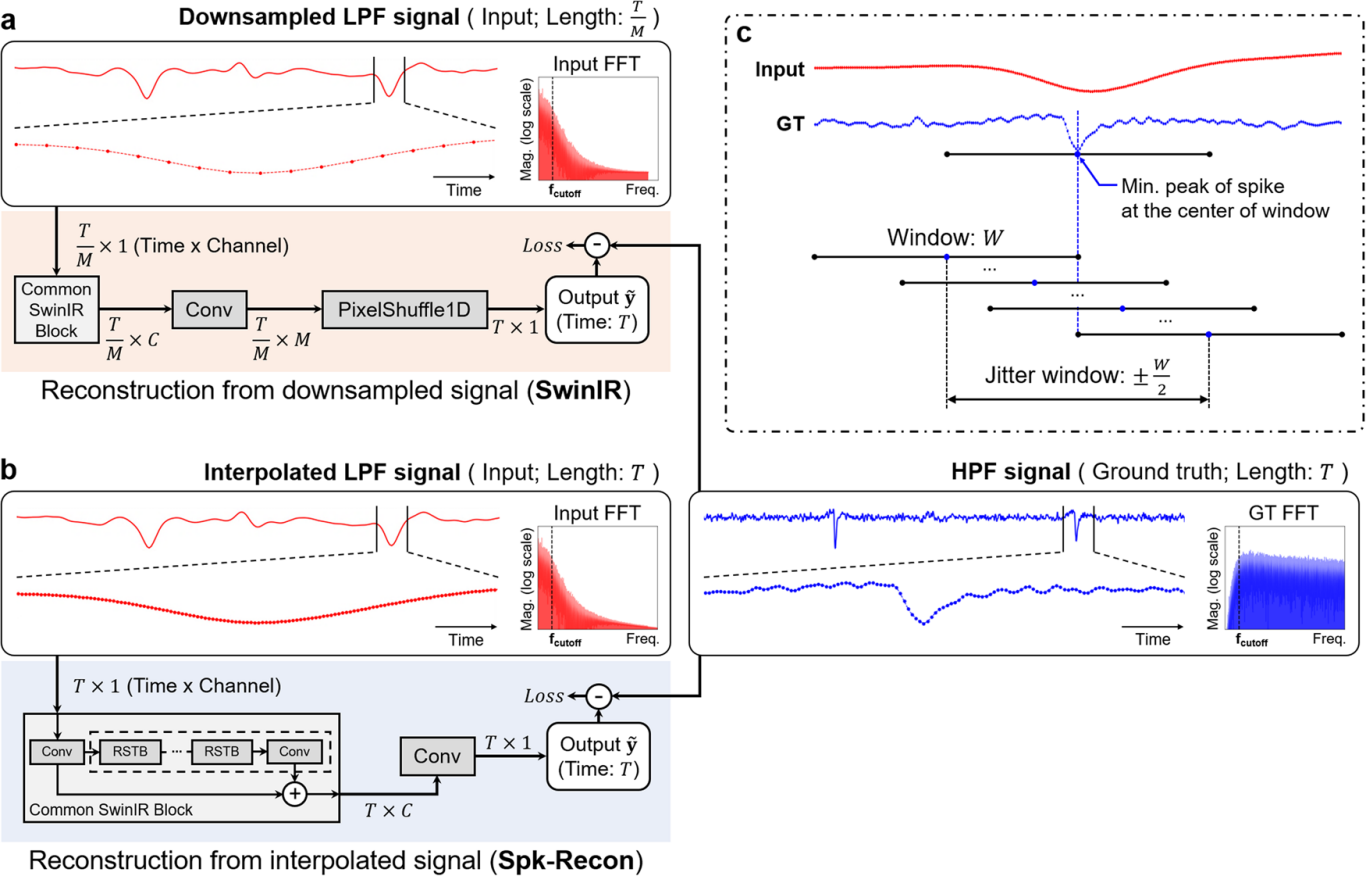
Schematics of Spk-Recon model architectures and training. **a** SwinIR model architecture for the downsampled low-pass filtered (LPF) input signal. Conv: convolutional layer. **b** Spk-Recon model architecture for the interpolated LPF input signal. RSTB residual Swin transformer block. **c** Spike-focused window selection for model training. The window size $W$ of the Spk-Recon was set to 128 data points, which is 5.12 ms.

The model architecture of the Spk-Recon is based on an image restoration transformer model, SwinIR^28^, consisting of the multi-head self-attention-based Swin transformer^29^ (Fig. 2). In the SwinIR, illustrated in Fig. 2a, the downsampled LPF signal (low-resolution data) is directly sent to the model as an input, and its resolution is increased by an upsampling block at the end of the model network. On the other hand, in our Spk-Recon, the temporal resolution of the downsampled LPF signal is enhanced in advance via the pre-interpolation process, and then the interpolated input is fed into the model. Thus, as depicted in Fig. 2b, unlike the SwinIR, the Spk-Recon comprises consecutive residual Swin transformer blocks (RSTBs) without a layer for upsampling. In addition, we devised a window selection method of model training focusing on neuronal spikes for improved reconstruction performance: spike-focused window selection (SFWS) (Fig. 2c). Spiking events generate within a short period (about a few ms) and occur sparsely. For this reason, if a training batch is randomly selected, many windows would not contain spikes, resulting in inefficient learning of the spike features. To achieve a more accurate restoration of spike information, half of each training batch was selected around spikes so that the windows (5.12 ms) always contain at least one spike, as shown in Fig. 2c. The details of network architecture and window selection are described in the Methods.

### Evaluation of Spk-Recon on in vitro neuronal datasets

**Fig. 3:**
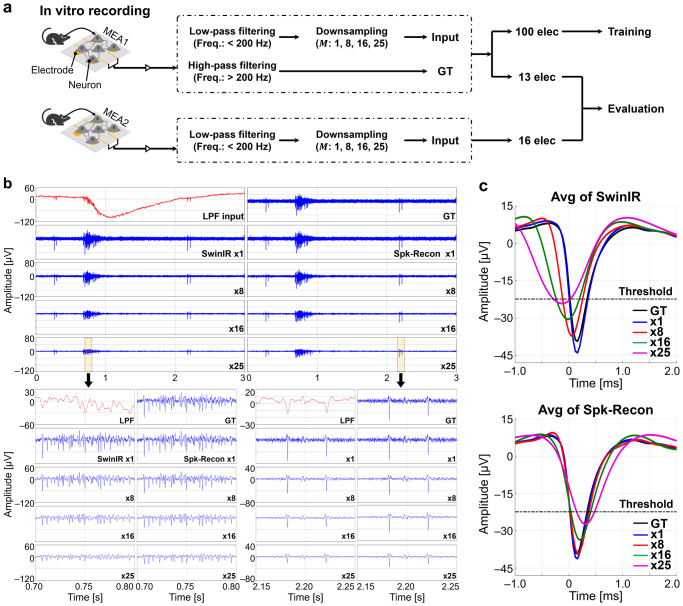
Signal reconstruction on in vitro datasets. **a** Signal processing for model training and evaluation. **b** Representative raw traces of low-pass filtered (LPF) input, ground truth (GT), and restored signals from a single electrode using SwinIR and Spk-Recon with different downsampling factors. Except for the LPF input signals, all others are plotted using the same scales. **c** Average waveforms of correctly restored spikes detected from the same electrode with (**b**).

To demonstrate the spike restoration ability of the Spk-Recon model, we applied the Spk-Recon to an in vitro neuronal dataset. Spontaneous electrical activities from in vitro rat hippocampal cultures were recorded using two MEAs, namely MEA1 and MEA2, with a sampling rate of 25 kHz (Fig. 3a and Supplementary Fig. 1). All signals were filtered by a low-pass filter (zero-phase fourth-order Butterworth filter, cutoff frequency of 200 Hz), followed by subsampling ($M$: 1, 8, 16, or 25). The downsampled LPF signals were re-upsampled using the Fourier method by the same factor ($L=M$) to obtain interpolated inputs for the Spk-Recon. In implementing our algorithm, the computation time for this pre-interpolation was much shorter than that for signal reconstruction from the Spk-Recon model (pre-interpolation: 0.09 ms vs. signal reconstruction: 36.29 ms; mean computation time over 300 repetitions for a single data sequence with a downsampling factor of 8), having little impact on total running time. The original high-sampled recording signals were high-pass filtered (HPF) with a 200 Hz cutoff frequency and were used as the GTs of spike reconstruction. For model training, signal pairs of LPF inputs and HPF GTs from 100 electrodes of the MEA1 were utilized. Those from the other 13 electrodes of the MEA1 and 16 electrodes of the MEA2 were applied only for evaluation.

We first restored high-frequency and high-resolution signals from LPF input signals of the MEA1 using the SwinIR and Spk-Recon with different downsampling factors. As shown in the representative raw traces of a single electrode in Fig. 3b, both ML models successfully reconstructed all the voltage fluctuations, including spiking events and even noise signals, in both time windows exhibiting burst behavior and tonic firing. The overall signal amplitudes decreased as the downsampling factor increased in both models. Figure 3c presents the average waveforms of the correctly restored spikes detected from the same electrode for each downsampling factor. Neuronal spikes were detected by setting the threshold of –6 standard deviation (SD) of the noise level of the GT signal. Among the reconstructed spikes, the spike timestamps within ±500 μs of GT spike timestamps were considered to be correctly restored in timing. The average waveforms of the two models shifted proportionally to the downsampling factor, but the outputs of the Spk-Recon were much less distorted than those of the SwinIR. In particular, there was no time delay of spike timestamps in the case of the Spk-Recon waveforms in Fig. 3c up to the downsampling factor of 16. For the multiple electrodes of the MEA1, the mean time delays of spike timestamps were –7.39 ± 2.05 μs, 10.80 ± 5.22 μs, 23.88 ± 23.08 μs (mean ± SD, *n* = 13 electrodes) for downsampling factors of 1, 8, and 16, respectively, which were smaller than the sampling period (40 μs) of the high-resolution signals.

**Fig. 4:**
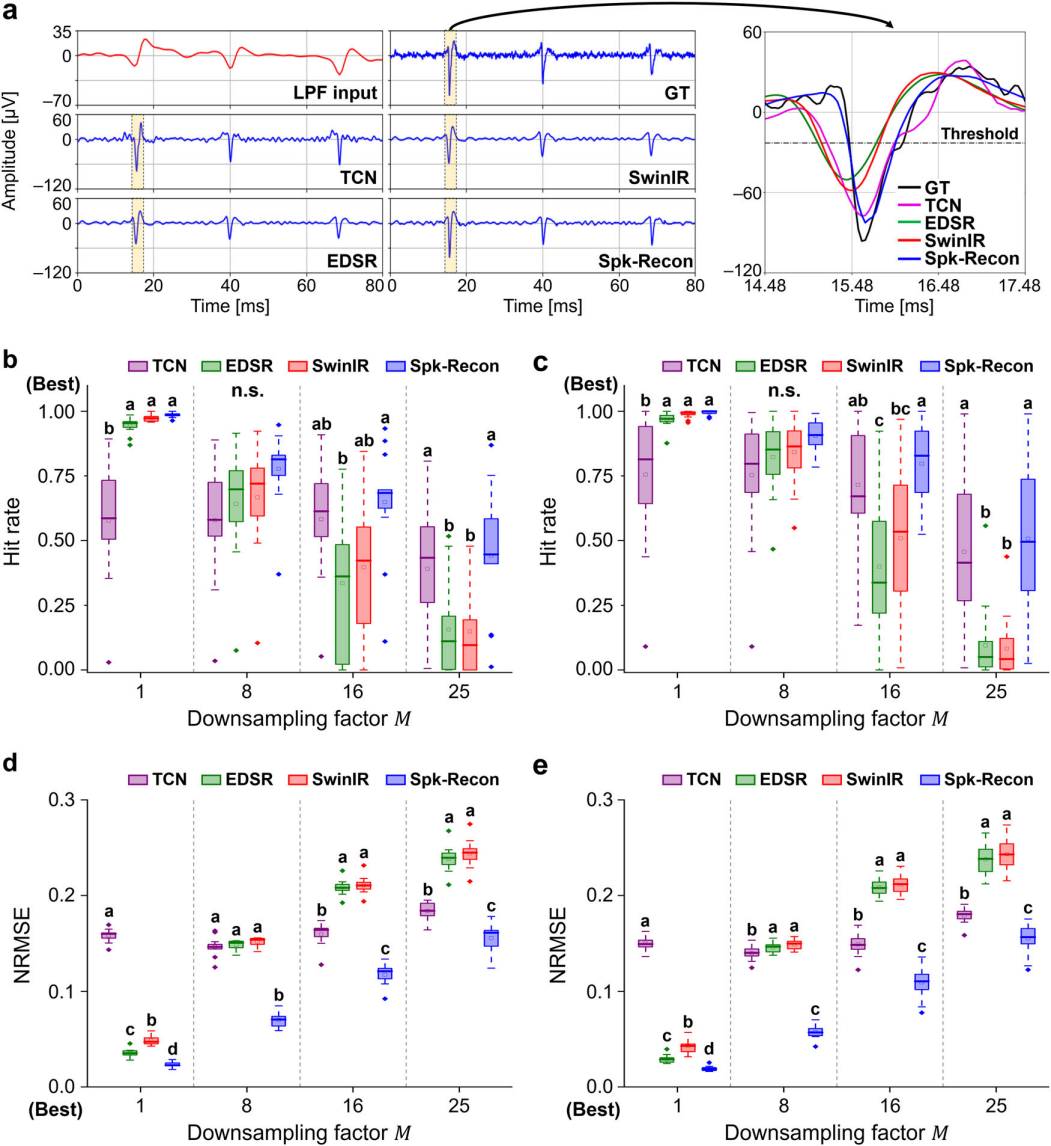
Model validation on an unseen dataset and comparison with CNN-based models. **a** Restored signals of the MEA2 with the downsampling factor of 16 and enlarged spike waveforms of the time window highlighted in the raw traces. Except for the low-pass filtered (LPF) input signal, all others are plotted using the same scale. **b** Hit rate of spike detection on the MEA1 with the different models. Note that a hit rate of 1 means that all the ground truth (GT) spikes are correctly reconstructed in timing. **c** Hit rate of the MEA2 with the different models. **d** Normalized root mean square error (NRMSE) between the restored and actual waveforms of the MEA1. Note that an NRMSE of 0 means that spike waveforms are perfectly restored. **e** NRMSE of the MEA2. The box represents the interquartile range (IQR), with median and mean values indicated by a horizontal line and ‘▫’, respectively. The whiskers extend to 1.5 times the IQR. One-way ANOVA with Tukey’s post-hoc test. *n* = 13 and 16 electrodes for MEA1 and MEA2, respectively. Source data are provided as a Source Data file.

We next applied the Spk-Recon on the MEA2 dataset, which was not used for the model training, to see if the spikes restoration could be done at a different neuronal culture. In addition, we also compared the performance of our transformer-based model against convolutional neural network (CNN)-based models: a temporal convolutional network (TCN)^30^ and an enhanced deep super-resolution network (EDSR)-Baseline^31^. Figure 4a shows representative reconstructed signals using four different ML models with a downsampling factor 16. Among the models, the Spk-Recon restored the most accurate spikes in occurrence times and waveforms. To quantify the spike reconstruction performance, we calculated a hit rate of detected spikes and a normalized root mean square error (NRMSE) of the waveforms. The hit rate is defined as the ratio of the number of correctly restored spikes (true positive) to that of GT spikes (true positive + false negative). The NRMSE of waveforms is computed through a point-by-point comparison of time windows from –1 to 2 ms of the GT timestamps and normalization by the peak-to-peak amplitude. As shown in Fig. 4b, c, the Spk-Recon showed the highest mean hit rates in all factors for the datasets from both MEAs. The mean values of the MEA1 were 0.99 ± 0.01, 0.78 ± 0.14, 0.65 ± 0.21, and 0.44 ± 0.24 (mean ± SD, *n* = 13 electrodes) for the downsampling factors of 1, 8, 16, and 25, respectively (Fig. 4b). Those of the MEA2 were 1.00 ± 0.01, 0.91 ± 0.06, 0.80 ± 0.16, and 0.51 ± 0.30 (*n* = 16 electrodes) for the downsampling factors of 1, 8, 16, and 25, respectively (Fig. 4c). As shown in Fig. 4d, e, the NRMSE values of the Spk-Recon were significantly lower than those of all the other models in the entire condition. The mean NRMSE values of the MEA1 were 0.02, 0.07, 0.12, and 0.16 (Fig. 4d), and those of the MEA2 were 0.02, 0.06, 0.11, and 0.16 (Fig. 4e) for the downsampling factors of 1, 8, 16, and 25, respectively. Additionally, we figured out the precision of spike detection (Supplementary Table 1), which is defined as the ratio of the number of correctly restored spikes (true positive) to that of spikes detected in the reconstructed signal (true positive + false positive). The mean precision values of the Spk-Recon were high in all conditions, ranging from 0.89 to 0.97, implying that most detected spikes occurred at correct timings. Taken together, the results demonstrated that the Spk-Recon achieved the best performance in reconstructing the accurate spike timings and waveforms on both MEA datasets. In particular, for the downsampling factor of 1, 16, and 25, the hit rates of Spk-Recon were significantly higher than those of CNN-based TCN and EDSR or transformer-based SwinIR. Moreover, the Spk-Recon showed significant improvement in reconstructing spike waveforms compared to the other models across all the downsampling factors.

**Fig. 5:**
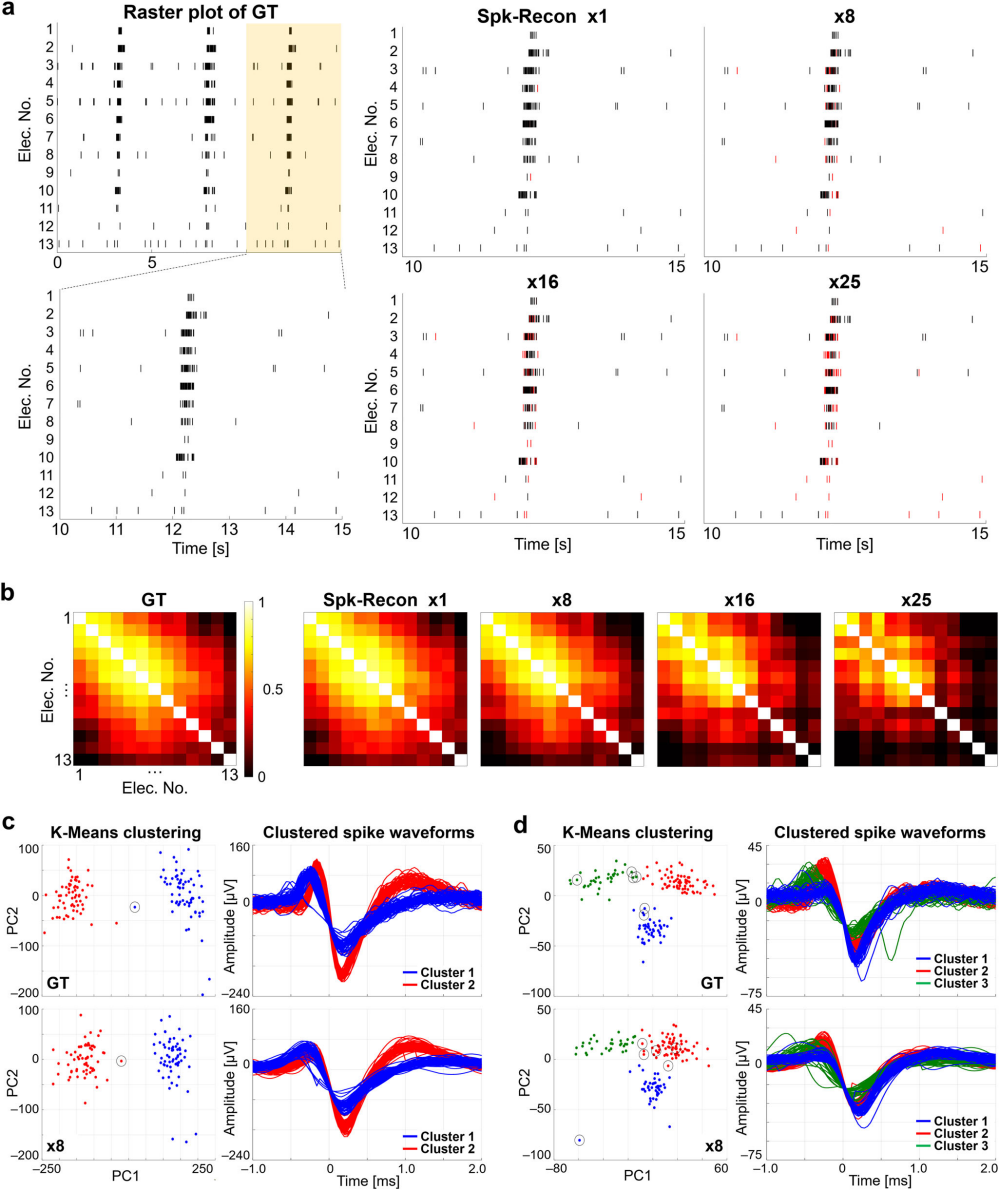
Neuronal spike train analysis. **a** Raster plots of ground truth (GT) and restored spikes using Spk-Recon with different downsampling factors. Red timestamps represent missing spikes. **b** Correlation coefficient matrices between spike trains from multiple electrodes. **c** Spike sorting of GT and restored spikes with a downsampling factor of 8 by principal component analysis-based feature extraction and K-Means clustering (2-cluster case). A black circle in the principal component space (PC1 vs. PC2) shows an incorrectly classified spike. Clustering accuracy of the reconstructed spikes: 99.23% (129/130 spikes). **d** Spike sorting (3-cluster case). Black circles in the principal component space (PC1 vs. PC2) show incorrectly grouped spikes. Clustering accuracy of the reconstructed spikes: 96% (144/150 spikes).

Using multichannel spikes reconstructed via the Spk-Recon, we assessed how the spatiotemporal information is restored for BMI systems: functional connectivity analysis and spike sorting. Figure 5a shows raster plots for different downsampling factors. The number of missing spikes, marked with red stamps, increased as the downsampling factor rose in overall electrodes, which is consistent with the hit rate reduction shown in Fig. 4b, c. To examine the functional connectivity of the neuronal networks, Pearson correlation coefficients for all spike train pairs of multiple electrodes were computed^32^, and the correlation matrices were constructed (Fig. 5b). Despite some missing spikes and slightly shifted timestamps, the correlation matrices for the downsampling factors up to 8 were similar to that of the GT without significant differences in the coefficients (*p* = 0.7243, 0.1542, 0.0131, and <0.0001 for the factor 1, 8, 16, and 25, respectively; Two-tailed two-sample *t* test for the coefficients compared with those of the GT), indicating that the spatial network connectivity was reasonably well re-established. This accurate restoration ability is essential for detecting significant changes in functional connectivity to identify differences in brain states between normal and pathological conditions^33,34^ or to clarify the effect of external stimulation and manipulation on network connectivity^35,36^.

Next, we performed spike sorting of the reconstructed spikes with a downsampling factor of 8 through principal component analysis-based feature extraction and K-Means clustering^37^. Figure 5c presents the clustering result of the GT and reconstructed spikes from a single electrode with two clusters. The spikes sorted into different clusters were visualized with different colors in the principal component (PC) space and waveform plots. All the spikes were correctly clustered except one (black circled in the PC space), showing an accuracy of 99.23% (129/130 spikes). The spike clustering result for a 3-cluster case (Fig. 5d) also achieved a high clustering accuracy of 96% (144/150 spikes), with only a few spikes incorrectly grouped. As a result, we found that the Spk-Recon model could reasonably restore the intrinsic information contained in network connectivity and waveform features, implying good applicability to advanced BMIs.

**Fig. 6:**
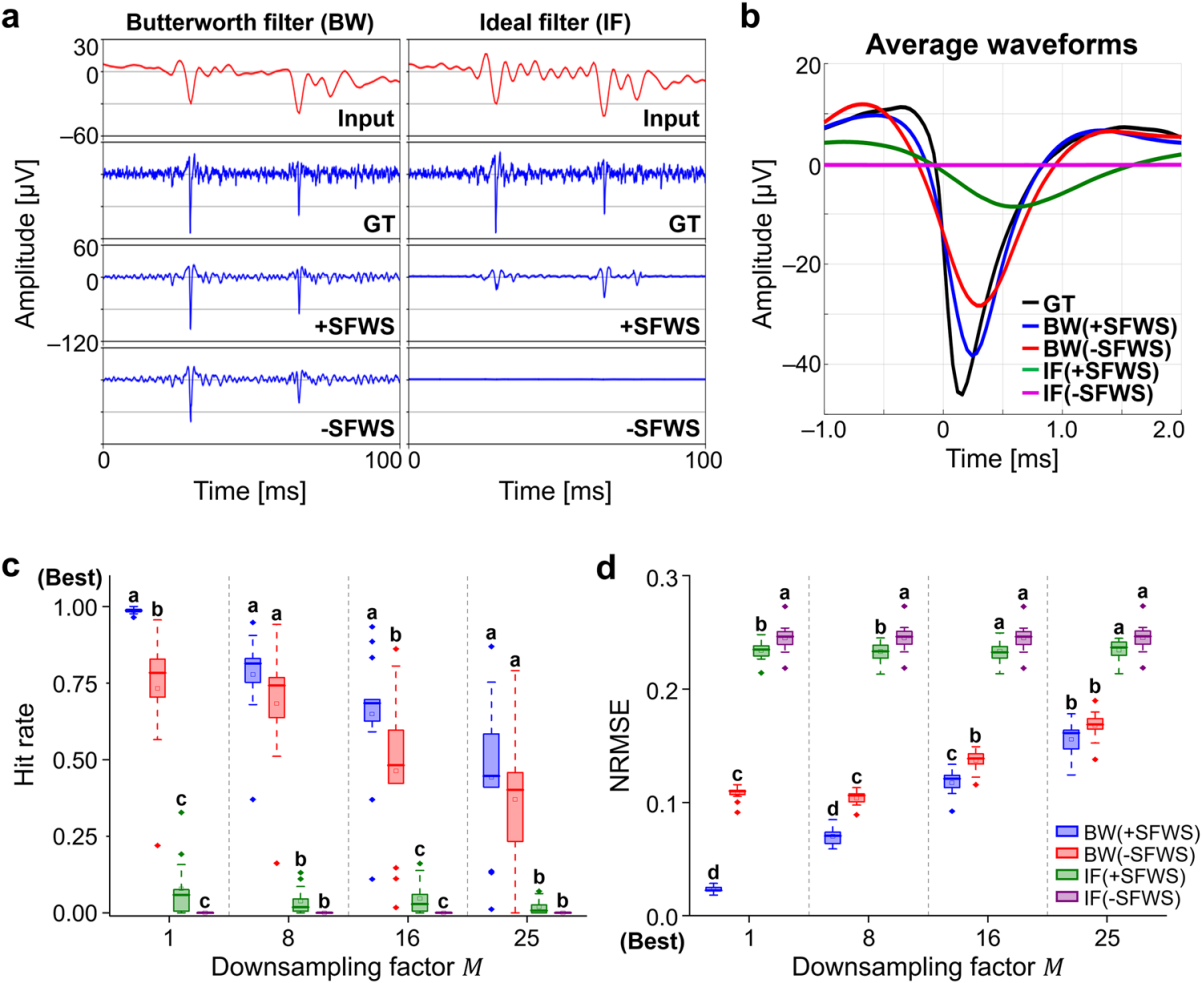
Ablation study results. **a** Raw traces and (**b**) average waveform profiles of restored outputs from the signals processed by the Butterworth filter (BW) or ideal filter (IF). The spike reconstruction was conducted with a downsampling factor of 16 with or without spike-focused window selection (+SFWS and -SFWS, respectively). Except for the low-pass filtered (LPF) input signals, all others are plotted using the same scale. **c** Hit rate and (**d**) normalized root mean square error (NRMSE) for different ablated conditions. Note that a hit rate of 1 means that all the ground truth (GT) spikes are correctly reconstructed in timing, and an NRMSE of 0 means that spike waveforms are perfectly restored. The box represents the interquartile range (IQR), with median and mean values indicated by a horizontal line and ‘▫’, respectively. The whiskers extend to 1.5 times the IQR. One-way ANOVA with Tukey’s post-hoc test. *n* = 13 electrodes. Source data are provided as a Source Data file.

To better understand how the Spk-Recon is able to reconstruct high-frequency neuronal spikes, we next performed two ablation experiments for Butterworth filtering (BW)-based signal processing and SFWS. First, we hypothesized that our model mainly functions to recover spike information from the residual high-frequency components in the LPF input signals. In our approach, the LPF inputs were processed using a Butterworth filter, which is a non-ideal filter, and this inevitably made that frequency components above the cutoff frequency were not completely removed (Fig. 2a, b and Supplementary Fig. 2). To ablate this effect, we used a dataset of input and GT signals, whose frequency bands were completely split through the ideal filtering (IF). Figure 6a, b show the restored signals of the downsampling factor of 16 with the realistic Butterworth filtering (BW( + SFWS)) and the ideal filtering (IF( + SFWS)). The amplitude of the signals in the IF case was much smaller than that of the BW case (a reduction of 77.67% in zero-to-peak amplitude), with a larger time delay (time delay from the minimum peak of GT; BW( + SFWS): 80 μs vs. IF( + SFWS): 480 μs). In the quantitative results, the reconstructed outputs of the IF case (IF( + SFWS)) showed an extensive reduction of the hit rate (Fig. 6c) and greater error of the signals (Fig. 6d) for all downsampling factors.

Next, we evaluated the effect of the SFWS on the reconstruction performance. As described in the previous section, the minibatch for model training was set so that half of them included at least one spike within their window (Fig. 2c). To remove this effect, we chose the batch by randomly picking out windows across the entire time series (-SFWS). Using the Butterworth filtering (BW(-SFWS) in Fig. 6a, b) resulted in a 25.97% decrease in the zero-to-peak amplitude compared to the BW( + SFWS) in the downsampling factor 16. Moreover, the training without the SFWS on the dataset produced by ideal filtering (IF(-SFWS)) causes a dramatic failure to recover signals. As shown in Fig. 6c, d, quantitative performance degradations were observed across all downsampling factors. To sum up, we confirmed that the residual high-frequency components in the LPF inputs highly enhanced overall signal reconstruction performance. The model training with the SFWS improved the restoration capability, especially regarding accurate spike waveforms.

### Applying Spk-Recon to in vivo neuronal datasets

**Fig. 7:**
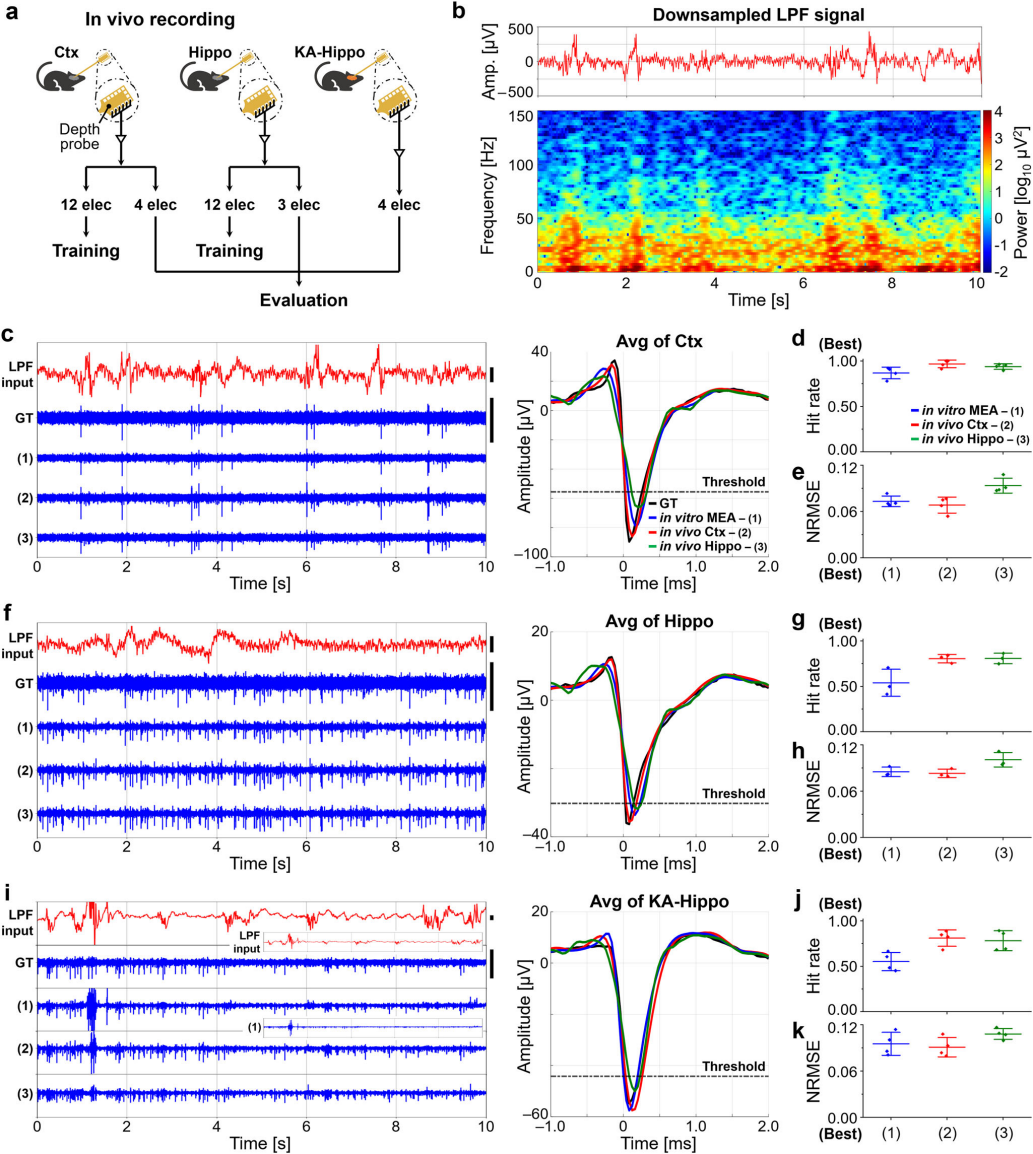
Signal restoration on in vivo datasets with the downsampling factor of 8. **a** Signal processing of cortex (Ctx), hippocampus (Hippo), and KA-treated mouse hippocampus (KA-Hippo) datasets for model training and evaluation. **b** Raw trace and spectrogram of the downsampled low-pass filtered (LPF) signal obtained from the Ctx before the interpolation process. **c** Reconstructed Ctx signals and their average spike waveforms using the Spk-Recon models trained on three different datasets (Trained on (1) in vitro MEA, (2) in vivo Ctx, or (3) in vivo Hippo datasets). Except for the LPF input signals, all other signals are plotted using the same scales. Scale bar: 200 μV. **d** Hit rate of spike detection and (**e**) normalized root mean square error (NRMSE) between the restored and actual waveforms of the Ctx dataset. *n* = 4 electrodes. Note that a hit rate of 1 means that all the ground truth (GT) spikes are correctly reconstructed in timing, and an NRMSE of 0 means that spike waveforms are perfectly restored. **f** Reconstructed Hippo signals and their average spike waveforms. Except for the LPF input signals, all other signals are plotted using the same scales. Scale bar: 100 μV. **g** Hit rate of spike detection and (**h**) NRMSE between the restored and actual waveforms of the Hippo dataset. *n* = 3 electrodes. **i** Reconstructed KA-Hippo signals of the KA-treated mouse and their average spike waveforms. Except for the LPF input signals, all other signals are plotted using the same scales. Scale bar: 200 μV. Insets display the same traces of the LPF input and the restored signal (1) on different y-axis scales (y-axis of the LPF input: –2–2 mV; y-axis of the restored signal (1): –500−400 μV). **j** Hit rate of spike detection and (**k**) NRMSE between the restored and actual waveforms of the KA-Hippo dataset. *n* = 4 electrodes. Data are presented as mean ± SD. Source data are provided as a Source Data file.

Finally, we applied the Spk-Recon to the datasets collected from mouse brains to investigate the applicability of our model to in vivo datasets. We measured neuronal signals from the cortex (Ctx) and hippocampus (Hippo) of anesthetized mice using a penetrating depth probe with 16 electrodes at a sampling rate of 25 kHz (Fig. 7a). The in vivo datasets were processed by low-pass filtering, downsampling, and pre-interpolation with a downsampling factor of 8 in the same way as the in vitro signals. Signal pairs of LPF inputs and HPF GTs from 12 electrodes of each recording were used for model training, and those of the other 4 or 3 electrodes from Ctx and Hippo recordings, respectively, were utilized for evaluation. Figure 7b presents a LPF and downsampled signal of spontaneous activity measured from the Ctx and a time-frequency spectrogram obtained from the signal. Using the downsampled LPF signal acquired in our proposed approach, temporal and spectral information of brain activity can be examined, especially in the low-frequency band covering typical LFPs, while significantly reducing the recording data volume. In Fig. 7b, it showed relatively high LFP power at low frequencies (<40 Hz) for the entire 10 s, and transient power increases in both low and higher frequency bands (40–100 Hz) at times when large voltage fluctuations occurred.

We restored high-frequency neuronal activity from the downsampled LPF signals of the Ctx and Hippo through the pre-interpolation process and reconstruction by the Spk-Recon model. We used three Spk-Recon models trained on different datasets: (1) in vitro MEA, (2) in vivo Ctx, and (3) in vivo Hippo datasets. As shown in the raw traces and average spike waveforms (Fig. 7c), the high-frequency signals of the Ctx test dataset exhibiting spiking events were well reconstructed, regardless of the training datasets. The reconstruction performances for the Ctx test dataset were comparable to the in vitro test results (Fig. 4), having similar ranges of the hit rate and NRMSE. The hit rates for the Spk-Recon models trained on in vitro MEA, in vivo Ctx, and in vivo Hippo datasets were 0.87 ± 0.06, 0.97 ± 0.04, and 0.94 ± 0.03 (mean ± SD, *n* = 4 electrodes), respectively (Fig. 7d). The mean NRMSE values were 0.07, 0.07, and 0.09 for in vitro MEA, in vivo Ctx, and in vivo Hippo datasets, respectively (Fig. 7e). In the reconstruction results of the Hippo test dataset showing continuous and frequent spiking activity (Fig. 7f), the overall signal traces and spike waveforms were restored similarly to that of GT; however, the hit rate using the model trained on in vitro MEA dataset was 0.54 ± 0.15, which is much lower than the model trained on in vivo datasets that were 0.80 ± 0.05 and 0.81 ± 0.06 for in vivo Ctx and in vivo Hippo, respectively (*n* = 3 electrodes) (Fig. 7g). This implies that the model trained under the in vivo condition performs better at the signal reconstruction of in vivo recordings, which are typically noisier than those from in vitro. The mean NRMSE values were 0.09, 0.08, and 0.10 for in vitro MEA, in vivo Ctx, and in vivo Hippo datasets, respectively (Fig. 7h).

Next, to examine whether the Spk-Recon is also applicable to pathological conditions, we acquired the neuronal signals from an anesthetized mouse in which seizure was induced via acute kainic acid (KA) injection (Fig. 7a). We recorded the signals in the hippocampus (KA-Hippo), which is known as the site of seizure induction in the KA-treated mouse^38^. LPF inputs of 4 electrodes were fed into the pre-trained model on in vitro MEA, in vivo Ctx, and in vivo Hippo datasets. Figure 7i shows the restored signals, representing similar traces and waveforms to those of the GT signal, but there was a noticeable error, especially in the Spk-Recon trained on the in vitro dataset, at the time when the LPF input greatly fluctuated over 1 mV (1–2 s in Fig. 7i). Consistent with the Hippo result under the condition without KA injection, the hit rate using the Spk-Recon trained on the in vitro dataset was 0.55 ± 0.10 (*n* = 4 electrodes) (Fig. 7j). The hit rates were highly improved by training the Spk-Recon model with the in vivo Ctx and Hippo datasets that were 0.81 ± 0.09 and 0.78 ± 0.11, respectively (Fig. 7j), comparable to the reconstruction performance on the in vitro test datasets (Fig. 4b, c). The mean NRMSE values ranged from 0.09 to 0.11, which were sufficiently low (Fig. 7k). Taken together, we have successfully demonstrated the broad applicability of the Spk-Recon, which allowed spike reconstruction on both in vitro and in vivo datasets from different brain regions, and even from the seizure-induced disease animal model.

## Discussion

In this study, we have developed an ML framework, the Spk-Recon model coupled with the pre-interpolation, to reconstruct neuronal spikes with high temporal resolution from downsampled lower-frequency neural recordings. By evaluating the reconstruction performance on multichannel neural datasets, we demonstrated the superiority of the Spk-Recon model in restoring high-resolution spikes from significantly downsampled signals with accurate spike timings and waveforms. The reconstructed spikes maintained the spatiotemporal information, resulting in comparable functional connectivity and spike sorting outcomes to the conventionally high-sampled HPF GT signals. We further showed that our trained Spk-Recon model worked well not only on in vitro datasets but also on in vivo datasets from different brain regions (cortex and hippocampus) and different pathological conditions (healthy and seizure-induced), empirically implying the feasibility and generality of our proposed framework. Although we used subsampled LPF inputs initially recorded at a high sampling rate, followed by low-pass filtering and downsampling, for comparison with the conventional high-sampled HPF GT spikes, the pre-trained model would be applied to low-frequency signals directly acquired on recording hardware at a low sampling rate in practical situations.

Our Spk-Recon-based restoration can simultaneously acquire abundant neuronal signals over a wide frequency range from LFPs to spikes: low-frequency band LFPs by direct recording at a low sampling rate and high-frequency band spikes by ML-based reconstruction. Previous works in data reduction techniques limitedly focused on obtaining only high-frequency bands of spikes^14–20^. Although there have been several studies to estimate spikes from low-frequency band LFPs using linear or nonlinear models^39–43^, they were still at the level of only inferring firing rates or obtaining spike timings, not the entire spike waveform characteristics. Being capable of taking both LFPs and spikes, our method can provide richer neuronal information that facilitates the analyses of LPF-spike correlations^44–47^ or brain functional connectivity^48–50^ based on spike-triggered averaging of LFPs. In particular, the ability to even restore waveform features with the high temporal resolution required for spike sorting would be useful for developing advanced BMIs using both LFPs and single-unit spikes^51–53^. All of these can be achieved with low recording data volume by conventional neural recording hardware.

From a hardware application point of view, our method of reducing the volume of neuronal data has high universality. We acquired the low-frequency band signals with uniformly lower sampling rates by a typical neural recording hardware and restored the high-sampling spikes through the ML-based software. That is, the signal acquisition with the reduced data volume for applying our model can be implemented in various commercial or customized systems of multichannel neural recording without additional hardware modification. Moreover, considering the recent efforts to increase the number of recording electrodes^4–6^, it would allow the collection of neuronal signals from more electrodes or for a longer duration within the same hardware resources. We expect it opens a new direction in developing next-generation BMIs capable of more in-depth analysis and control of brain functions with reduced hardware resources and minimal thermal tissue damage.

Lastly, the Spk-Recon can provide a versatile framework for signal estimation between various types of neural recordings. The results with the downsampling factor 1 in Fig. 3b demonstrated the function of our framework in restoring high-frequency information from lower-frequency band signals. This capability could be applied to neuronal activities measured with different recording modalities like intracortical recording, electrocorticography (ECoG), and electroencephalography (EEG). For example, low-frequency to high-frequency signal restoration could be possible from ECoG to spike or from EEG to ECoG. In addition, by utilizing the latest MEA technologies that enable network-wide intracellular recording^54^, the reconstruction of intracellular neuronal signals, such as action potentials or subthreshold synaptic signals, from extracellular recordings could also be achieved. This could enable even higher performance in BMIs by developing new systems based on multimodal or multiscale signals with less physical invasiveness^55,56^.

## Methods

### Neuronal dataset acquisition and processing

All experimental procedures were approved by the Institutional Animal Care and Use Committee (IACUC) of Daegu Gyeongbuk Institute of Science and Technology (DGIST), and all experiments were performed in accordance with the guidance of the IACUC of DGIST (DGIST-IACUC-22102605-0004).

We acquired in vitro neuronal signals from dissociated cultures using MEAs with 120 electrodes (120MEA100/30iR-ITO, 120MEA200/30iR-Ti; Multi Channel Systems, Germany). Before cell seeding, the surface of the MEAs was coated with 0.05 mg/mL of poly-D-lysine (A3890401; Gibco; Thermo Fisher Scientific, MA, USA) diluted in Dulbecco’s Phosphate-Buffered Saline (LB001-02; WELGENE, South Korea) to make it cell-adhesive. Rat hippocampi isolated from embryonic day 18 Sprague-Dawley rats (DBL, South Korea) were dissociated in Hank’s buffer salt solution (LB003-02; WELGENE), and the cell pellet was obtained by centrifugation. The pellet was resuspended in a medium composed of Neurobasal medium (21103049; Gibco), B-27 supplement (17504044; Gibco), 2 mM GlutaMAX supplement (35050061; Gibco), 1% penicillin-streptomycin (15140122; Gibco), and 12.5 μM L-glutamine (25030081; Gibco), followed by neuron seeding on the MEAs with the density of 1000 cells/mm^2^. Two weeks after the cultivation in an incubator at 37 °C and 5% CO_2_, the spontaneous activities of the cultured neurons were measured from multiple electrodes and sampled at 25 kHz by a DAQ card (band-pass filter: 0.1 Hz−3.5 kHz; MEA2100-Mini-Systems; Multi Channel Systems).

We collected in vivo neuronal datasets from three C57BL/6 J mice using neural probes that have 16 electrodes (A1x16−3 mm-50-703; NeuroNexus, MI, USA). Mice, born and reared in standard mouse cages with food and water, were maintained at a temperature of 22 ± 1 °C and a humidity of 40–60% with a 12:12-h light/dark cycle at the DGIST animal facility. Male mice aged 11−12 weeks were used for the study, and all surgeries were carried out under aseptic conditions. The mice were anesthetized through intraperitoneal injection of urethane (1.5 g/kg) and placed in a stereotaxic apparatus (RWD Life Science, China) for acute recording. After incising the skin and drilling holes in the skull, the neural probe was implanted in the auditory cortex (AP –3 mm, ML + 3.83 mm, DV –2.5 mm) or hippocampus (AP –1.6 mm, ML + 1.6 mm, DV –1.7 mm). Reference and ground wires were inserted into the cerebellum. Particularly, for the recording in the seizure-induced hippocampus, kainic acid (10 mg/kg; K0250; Sigma-Aldrich, MA, USA) was treated for induction of seizures. Using a DAQ system (band-pass filter: 0.98 Hz–7.60 kHz with a notch filter of 60 Hz; RHS Stim/Recording System; Intan Technologies, CA, USA), signals were recorded at a sampling rate of 25 kHz.

Both in vitro and in vivo neuronal signals were separated into LPF input and HPF GT signals using zero-phase fourth-order Butterworth filters with a cutoff frequency of 200 Hz. Each signal pair was normalized to the maximum absolute values of the background noise of the HPF signal. To obtain downsampled and interpolated LPF inputs, LPF signals were subsampled by factors of 1, 8, 16, or 25 and then re-upsampled using the Fourier method^27^ by the same factor. All signal processing was performed in Python 3.8.8 using the SciPy library.

### Network architecture and implementation details

We constructed the Spk-Recon model based on a transformer-based SwinIR model^28^ (Common SwinIR Block in Fig. 2a, b), initially proposed for image restoration. Given an input signal ${{{{{{{\bf{I}}}}}}}}_{{{{{{{\bf{Spk}}}}}}}{{{{{{\boldsymbol{-}}}}}}}{{{{{{\bf{Recon}}}}}}}}$ with a length $T$, a shallow feature of the same length with $C$ channels is extracted by convolving the input signal with 1D kernels (${k}_{{SF}}\left(\cdot \right)\!:{{\mathbb{R}}}^{T\times 1}\to {{\mathbb{R}}}^{T\times C}$; First ‘Conv’ in Fig. 2b). By passing the feature through several consecutive RSTBs, each of which is composed of multi-head self-attention-based Swin transformer layers (STLs)^29^, followed by an additional convolution layer, a deep feature with the same size as the input feature is obtained (${k}_{{DF}}\left(\cdot \right):{{\mathbb{R}}}^{T\times C}\to {{\mathbb{R}}}^{T\times C}$). The shallow and deep features are combined with a skip connection.

Unlike the SwinIR, the input for the Spk-Recon is a pre-interpolated signal with the same temporal resolution as the output signal to be reconstructed. This results in a major difference in the last part of the network architecture: the absence of a layer for upsampling. The last layer of the Spk-Recon is a 1D convolution (${k}_{C}\left(\cdot \right):{{\mathbb{R}}}^{T\times C}\to {{\mathbb{R}}}^{T\times 1}$; Last ‘Conv’ in Fig. 2b) to generate a one-channel output signal $\widetilde{{{{{{{\bf{y}}}}}}}}$, replacing the sub-pixel convolution layer for upsampling in the SwinIR (‘PixelShuffle 1D’ in Fig. 2a). The final output signal $\widetilde{{{{{{{\bf{y}}}}}}}}$ is formulated as

1 $$\widetilde{{{{{{\bf{y}}}}}}}={k}_{C}({{{{{{\bf{F}}}}}}}_{{{{{{\bf{SF}}}}}}}+{{{{{{\bf{F}}}}}}}_{{{{{{\bf{DF}}}}}}}),$$

where $${{{{{{\bf{F}}}}}}}_{{{{{{\bf{SF}}}}}}}={k}_{{SF}}\left({{{{{{\bf{I}}}}}}}_{{{{{{\bf{Spk}}}}}}-{{{{{\bf{Recon}}}}}}}\right)$$ and $${{{{{{\bf{F}}}}}}}_{{{{{{\bf{DF}}}}}}}={k}_{{DF}}\left({{{{{{\bf{F}}}}}}}_{{{{{{\bf{SF}}}}}}}\right).$$

The input length and the kernel size of 1D convolution were set to 128 and 3 data points, respectively. The corresponding GT for supervised learning had the same length as the input, 128 data points. The number of feature channels, RSTBs, and STLs were 180, 6, and 6, the same as the previous study^28^. Different networks were trained in individual downsampling factors ($M$: 1, 8, 16, and 25) for 200 epochs with a batch size of 16. A mean squared error loss and an Adam optimizer with a fixed learning rate of 1e-4 were utilized for optimization. In the evaluation process, 128 data points were sequentially presented to the trained network by sliding the window by 64 data points.

As baseline models for comparison, we used transformer-based SwinIR^28^, CNN-based enhanced deep super-resolution network (EDSR)-Baseline^31^, and CNN-based TCN^30^. The input for the SwinIR and EDSR-Baseline was the downsampled LPF signals, whereas that of TCN was the pre-interpolated LPF signal, the same as the Spk-Recon. To make the numbers of parameters similar to our Spk-Recon, the hyperparameters of the baseline models were set as follows: SwinIR (input length: 128; GT length: 128$M$; kernel size: 3; the number of channels, RSTBs, STLs: 180, 6, 6), EDSR-Baseline (input length: 128; GT length: 128$M$; kernel size: 3; the number of channels, residual blocks: 262, 16), and TCN (input length: 127; GT length: 1; kernel size: 3; the number of channels, stacked blocks: 554, 6). All models were implemented in Python (Supplementary Code 1) using PyTorch 1.7.1 and were trained and evaluated on NVIDIA GeForce RTX 3090.

### Spike-focused window selection for model training using spike jittering

We constructed a minibatch with a batch size of $B$ and a GT window size of $W$: at least one spike is included in the window for the first half of the batch, and their minimum peak is placed on a random position within the window by jittering the spike timing as follows (Fig. 2c). Let us assume that a time series data ${{{{{{{\bf{y}}}}}}}}^{{{{{{{\bf{k}}}}}}}}\in {{\mathbb{R}}}^{N}$, which is a high-frequency and high-resolution signal from electrode $k$, has ${s}^{k}$ spikes ($k=1,\ldots,{K}$). For the $i$-th spike of the electrode, ${n}_{i}^{k}$ denotes the time point where the minimum peak of the spike waveform is located ($i=1,\ldots,{s}^{k}$). To select windows of the first half batch, we chose $\frac{B}{2}$ peaks ${n}_{{i}_{j}}^{{k}_{j}}$, by picking out the electrodes and their corresponding spikes {${k}_{j}$, ${i}_{j}$} ($j=1,\ldots,\frac{B}{2}$) and the equal number of jitters ${\tau }_{j}$ in the interval $\left(-\frac{W}{2}\right.,\,\left.\frac{W}{2}\right]$ at random. With the chosen variables, the data within the time interval $\left[{n}_{{i}_{j}}^{{k}_{j}}+{\tau }_{j}-\frac{W}{2}\right.,\,\left.{n}_{{i}_{j}}^{{k}_{j}}+{\tau }_{j}+\frac{W}{2}\right)$ is sampled as the $j$-th window. The other windows in the second half batch are randomly sampled using the time series data of $N$ time points from $K$ electrodes.

### Neuronal data analysis

We detected neuronal spikes by setting the threshold –6 SD of the background noise of the GT signals and identifying time points crossing the threshold as spike timestamps. The restored spikes whose timestamps were located within ±500 μs from GT spike timestamps were defined as correctly reconstructed.

To construct the correlation matrices for assessing functional connectivity (Fig. 5b), rate histograms with 50 ms-bin width were obtained for individual electrodes. Then, a Pearson correlation coefficient, an element of the correlation matrix, was computed between the rate histograms of each electrode pair. To sort the detected spikes (Fig. 5c, d), features of the spike waveforms were extracted by calculating principal component (PC) scores. For the clustering, the K-Means algorithm, in which the number of clusters was determined to be 2 or 3, was applied to the first two PCs (PC1 and PC2).

The time-frequency spectrogram (Fig. 7b) of the downsampled LPF signal, sampled at 3125 Hz, was generated by a short-time Fourier transform using a Hamming window of 1250 samples, an overlap of 1125 samples, and the number of FFT points of 616. All data analyses were carried out using MATLAB R2022b (MathWorks, MA, USA) (Supplementary Code 1), and all statistical data were plotted and tested using OriginPro 2021 (OriginLab, MA, USA).

### Reporting summary

Further information on research design is available in the Nature Portfolio Reporting Summary linked to this article.

### Supplementary information

Supplementary Information

Description of Additional Supplementary Files

Supplementary Code 1

Reporting Summary

Peer Review

### Source data

Source Data

## Data Availability

The test datasets of in vitro MEA, in vivo Ctx, in vivo Hippo, and in vivo KA-Hippo and the pre-trained models using in vitro MEA, in vivo Ctx, and in vivo Hippo training datasets generated in this study are available on Zenodo at https://zenodo.org/records/10113126^57^. The processed data supporting the findings of this study are available in the Source Data file. Source data are provided with this paper.

## Code availability

The custom Python and MATLAB codes for the machine learning and the data analyses reported in this study are provided in Supplementary Code 1.
