# Supplementary material for: Machine learning-based high-frequency neuronal spike reconstruction from low-frequency and low-sampling-rate recordings
Source: Nat Commun. 2024 Jan 20;15:635. doi: 10.1038/s41467-024-44794-2 (PMC10799928; doi:10.1038/s41467-024-44794-2)
Supplement: Supplementary file 1 — Supplementary Information [file 41467_2024_44794_MOESM1_ESM.pdf]

[Supplementary information]

Machine learning-based high-frequency neuronal spike reconstruction from low-frequency and low-sampling-rate recordings

Nari Hong[1,2], Boil Kim[3], Jaewon Lee[1,2], Han Kyoung Choe[3], Kyong Hwan Jin[1,4*], and Hongki Kang[1,2*]

1. Department of Electrical Engineering and Computer Science, Daegu Gyeongbuk Institute of Science and Technology (DGIST), Daegu 42988, Republic of Korea
2. Information and Communication Engineering Research Center, Daegu Gyeongbuk Institute of Science and Technology (DGIST), Daegu, 42988 Republic of Korea
3. Department of Brain Sciences, Daegu Gyeongbuk Institute of Science and Technology (DGIST), Daegu, 42988, Republic of Korea
4. School of Electrical Engineering, Korea University, Seoul, 02841, Republic of Korea

*Corresponding address: hkang@dgist.ac.kr, kyong_jin@korea.ac.kr

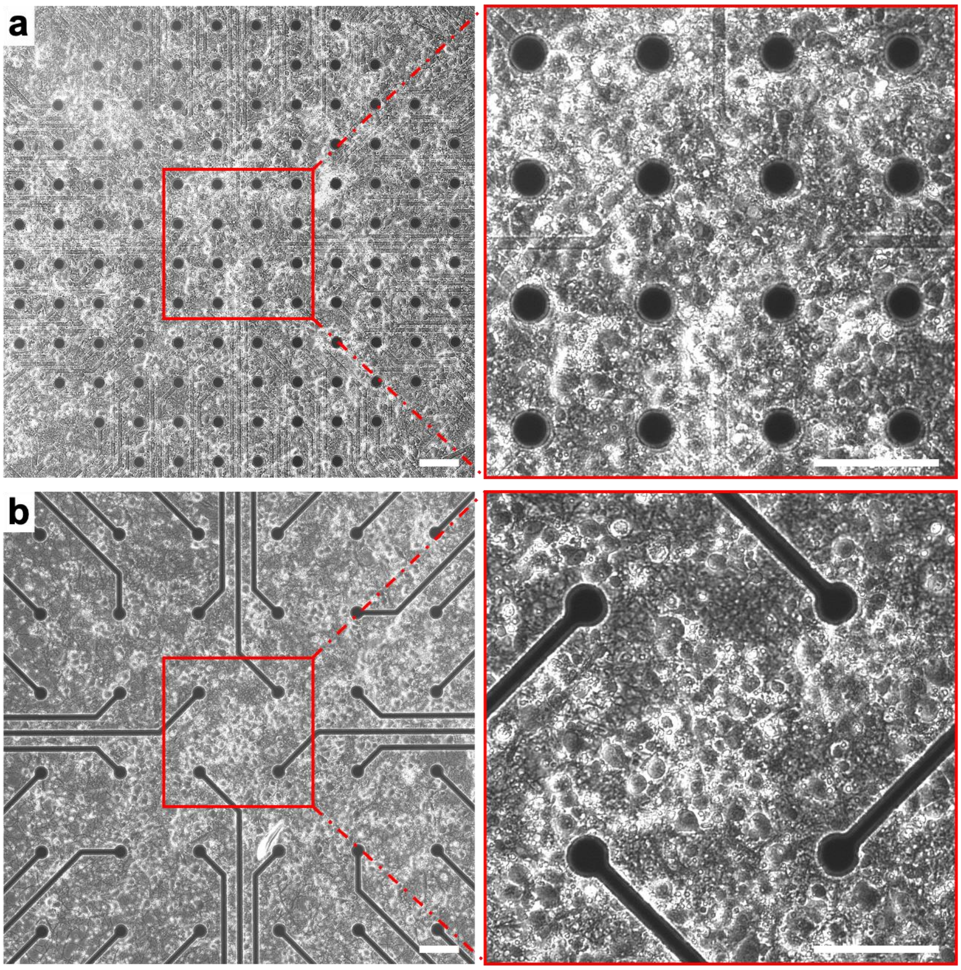

Supplementary Fig. 1 | In vitro neuronal networks cultured on MEAs for two weeks. a Phase-contrast images of the cultured neuronal network on the MEA1. **b** Phase-contrast images of the cultured neuronal network on the MEA2. Scale bar: 100 μm.

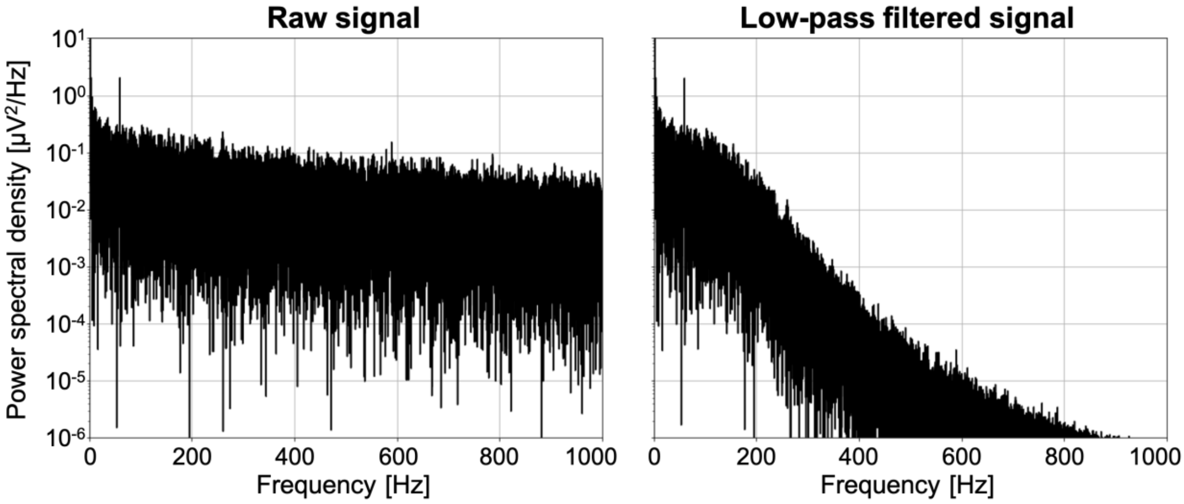

Supplementary Fig. 2 | Power spectral densities of raw and low-pass filtered signals.

Supplementary Table 1 | Precision of spike detection on two MEAs with different models and downsampling factors. The precision is defined as the ratio of the number of correctly restored spikes to that of spikes detected in the reconstructed signal. Data are presented as mean ± SD.

**Training dataset:** MEA1 (n = 100 electrodes)		**Downsampling factor M**			
**Test dataset**	**Model**	1	8	16	25
MEA1 (n = 13 electrodes)	TCN	0.91 ± 0.07	0.91 ± 0.07	0.88 ± 0.06	0.91 ± 0.08
	EDSR	0.98 ± 0.01	0.96 ± 0.04	0.94 ± 0.07	0.82 ± 0.15
	SwinIR	0.85 ± 0.07	0.96 ± 0.04	0.94 ± 0.07	0.77 ± 0.15
	Spk-Recon	0.93 ± 0.04	0.95 ± 0.03	0.89 ± 0.07	0.91 ± 0.08
MEA2 (n = 16 electrodes)	TCN	0.95 ± 0.05	0.94 ± 0.06	0.91 ± 0.06	0.94 ± 0.06
	EDSR	0.99 ± 0.01	0.98 ± 0.02	0.97 ± 0.04	0.91 ± 0.09
	SwinIR	0.90 ± 0.07	0.98 ± 0.02	0.98 ± 0.03	0.85 ± 0.12
	Spk-Recon	0.96 ± 0.03	0.97 ± 0.02	0.92 ± 0.07	0.92 ± 0.10
