# Supplementary material for: Machine learning-based high-frequency neuronal spike reconstruction from low-frequency and low-sampling-rate recordings
Source: Nat Commun. 2024 Jan 20;15:635. doi: 10.1038/s41467-024-44794-2 (PMC10799928; doi:10.1038/s41467-024-44794-2)
Supplement: Supplementary file 2 — Description of Additional Supplementary Files [file 41467_2024_44794_MOESM2_ESM.pdf]

Description of Additional Supplementary Files

File name: Supplementary Code 1

Description: Custom Python and MATLAB codes for the machine learning and the data analyses.
