# Supplementary material for: Machine learning-based high-frequency neuronal spike reconstruction from low-frequency and low-sampling-rate recordings
Source: Nat Commun. 2024 Jan 20;15:635. doi: 10.1038/s41467-024-44794-2 (PMC10799928; doi:10.1038/s41467-024-44794-2)
Supplement: Supplementary file 4 — Reporting Summary [file 41467_2024_44794_MOESM4_ESM.pdf]

nature portfolio

Corresponding author(s): Hongki Kang

Last updated by author(s): Nov 11, 2023

Reporting Summary

Nature Portfolio wishes to improve the reproducibility of the work that we publish. This form provides structure for consistency and transparency in reporting. For further information on Nature Portfolio policies, see our Editorial Policies and the Editorial Policy Checklist.

Statistics

For all statistical analyses, confirm that the following items are present in the figure legend, table legend, main text, or Methods section.

n/a	Confirmed	
☐	☒	The exact sample size (*n*) for each experimental group/condition, given as a discrete number and unit of measurement
☐	☒	A statement on whether measurements were taken from distinct samples or whether the same sample was measured repeatedly
☐	☒	The statistical test(s) used AND whether they are one- or two-sided<br>*Only common tests should be described solely by name; describe more complex techniques in the Methods section.*
☒	☐	A description of all covariates tested
☒	☐	A description of any assumptions or corrections, such as tests of normality and adjustment for multiple comparisons
☐	☒	A full description of the statistical parameters including central tendency (e.g. means) or other basic estimates (e.g. regression coefficient) AND variation (e.g. standard deviation) or associated estimates of uncertainty (e.g. confidence intervals)
☐	☒	For null hypothesis testing, the test statistic (e.g. *F*, *t*, *r*) with confidence intervals, effect sizes, degrees of freedom and *P* value noted<br>*Give P values as exact values whenever suitable.*
☒	☐	For Bayesian analysis, information on the choice of priors and Markov chain Monte Carlo settings
☒	☐	For hierarchical and complex designs, identification of the appropriate level for tests and full reporting of outcomes
☐	☒	Estimates of effect sizes (e.g. Cohen's *d*, Pearson's *r*), indicating how they were calculated

Our web collection on statistics for biologists contains articles on many of the points above.

Software and code

Policy information about availability of computer code

Data collection	Multi Channel Experimenter v2.16.0 (Multi Channel Systems) and RHX Data Acquisition Software v3.0 (Intan Technologies) were used for data collection.
Data analysis	Python 3.8.8 with SciPy library and PyTorch 1.7.1 were used for signal processing and model implementation.<br>MATLAB R2022b and OriginPro 2021 were used for data plotting, quantification, and statistical analysis.<br>The custom Python and MATLAB codes for the machine learning and the data analyses reported in this study are provided in Supplementary Code.

For manuscripts utilizing custom algorithms or software that are central to the research but not yet described in published literature, software must be made available to editors and reviewers. We strongly encourage code deposition in a community repository (e.g. GitHub). See the Nature Portfolio guidelines for submitting code & software for further information.

Data

Policy information about availability of data

All manuscripts must include a data availability statement. This statement should provide the following information, where applicable:

- Accession codes, unique identifiers, or web links for publicly available datasets
- A description of any restrictions on data availability
- For clinical datasets or third party data, please ensure that the statement adheres to our policy

The test datasets of in vitro MEA, in vivo Ctx, in vivo Hippo, and in vivo KA-Hippo and the pre-trained models using in vitro MEA, in vivo Ctx, and in vivo Hippo datasets generated in this study are available on Zenodo. The processed data supporting the findings of this study are available in the Source Data file. Source data are provided with this paper.

Research involving human participants, their data, or biological material

Policy information about studies with human participants or human data. See also policy information about sex, gender (identity/presentation), and sexual orientation and race, ethnicity and racism.

Reporting on sex and gender	N/A
Reporting on race, ethnicity, or other socially relevant groupings	N/A
Population characteristics	N/A
Recruitment	N/A
Ethics oversight	N/A

Note that full information on the approval of the study protocol must also be provided in the manuscript.

Field-specific reporting

Please select the one below that is the best fit for your research. If you are not sure, read the appropriate sections before making your selection.

☒ Life sciences ☐ Behavioural & social sciences ☐ Ecological, evolutionary & environmental sciences

For a reference copy of the document with all sections, see nature.com/documents/nr-reporting-summary-flat.pdf

Life sciences study design

All studies must disclose on these points even when the disclosure is negative.

Sample size	Sample sizes were determined based on the relevant published literature and by the nature of the experimental design.<br>For the in vitro test, a large number of samples were chosen for model training to improve the training efficiency, and the others (about 30 samples from two MEAs) were selected for model evaluation and statistical comparison. They were adequate as the difference between experimental groups was measurable and reproducible.<br>For the in vivo test, because the number of electrodes is relatively small compared to the in vitro test, the sample sizes were determined so that three-quarters of the samples were used for training, and the others were used for testing.<br>The sample sizes (n) are described in the figure legends.
Data exclusions	No data were excluded.
Replication	All experiments were performed in at least two biological replicates or animals. All attempts were successful.
Randomization	Rats for in vitro cell culture and mice for in vivo recording were randomly selected.
Blinding	Blinding was not relevant as data acquisition and analysis were automated using the programs.

Reporting for specific materials, systems and methods

We require information from authors about some types of materials, experimental systems and methods used in many studies. Here, indicate whether each material, system or method listed is relevant to your study. If you are not sure if a list item applies to your research, read the appropriate section before selecting a response.

Materials & experimental systems

n/a	Involved in the study
☒	☐ Antibodies
☒	☐ Eukaryotic cell lines
☒	☐ Palaeontology and archaeology
☐	☒ Animals and other organisms
☒	☐ Clinical data
☒	☐ Dual use research of concern
☒	☐ Plants

Methods

n/a	Involved in the study
☒	☐ ChIP-seq
☒	☐ Flow cytometry
☒	☐ MRI-based neuroimaging

Animals and other research organisms

Policy information about studies involving animals; ARRIVE guidelines recommended for reporting animal research, and Sex and Gender in Research

Laboratory animals	Sprague-Dawley rats (embryonic day 18) and C57BL/6J mice (11 - 12 weeks old) were used in this study. Mice, born and reared in standard mouse cages with food and water, were maintained at a temperature of 22 ± 1°C and a humidity of 40 – 60% with a 12:12-h light/dark cycle at the DGIST animal facility.
Wild animals	The study did not involve wild animals.
Reporting on sex	Rats of both sexes were randomly used for in vitro cell culture. Only male mice were used for in vivo recording.
Field-collected samples	The study did not involve samples collected from the field.
Ethics oversight	All experimental procedures were approved by the Institutional Animal Care and Use Committee (IACUC) of Daegu Gyeongbuk Institute of Science and Technology (DGIST), and all experiments were performed in accordance with the guidance of the IACUC of DGIST (DGIST-IACUC-22102605-0004).

Note that full information on the approval of the study protocol must also be provided in the manuscript.
