# Supplementary material for: Machine learning-based high-frequency neuronal spike reconstruction from low-frequency and low-sampling-rate recordings
Source: Nat Commun. 2024 Jan 20;15:635. doi: 10.1038/s41467-024-44794-2 (PMC10799928; doi:10.1038/s41467-024-44794-2)
Supplement: Supplementary file 5 — Peer Review [file 41467_2024_44794_MOESM5_ESM.pdf]

nature portfolio

Peer Review File

Machine learning-based high-frequency neuronal spike reconstruction from low-frequency and low-sampling-rate recordings

Open Access This file is licensed under a Creative Commons Attribution 4.0 International License, which permits use, sharing, adaptation, distribution and reproduction in any medium or format, as long as you give appropriate credit to the original author(s) and the source, provide a link to the Creative Commons license, and indicate if changes were made. In the cases where the authors are anonymous, such as is the case for the reports of anonymous peer reviewers, author attribution should be to 'Anonymous Referee' followed by a clear attribution to the source work. The images or other third party material in this file are included in the article's Creative Commons license, unless indicated otherwise in a credit line to the material. If material is not included in the article's Creative Commons license and your intended use is not permitted by statutory regulation or exceeds the permitted use, you will need to obtain permission directly from the copyright holder. To view a copy of this license, visit http://creativecommons.org/licenses/by/4.0/.

REVIEWER COMMENTS

Reviewer #1 (Remarks to the Author):

The authors of this manuscript introduced a machine-learning approach to reconstruct high-frequency neuronal spikes using low-sampled low-frequency band signals. Interestingly, their trained model can estimate high-frequency details of spiking activity, such as spike timing, waveform, and network connectivity, even when working with datasets that are down-sampled by a factor of eight. However, their approach and evaluation method may be where reconstruction is guaranteed only when using low-pass filtered and down-sampled data from the neuronal signals initially recorded with a wide frequency range at a high sampling rate. Thus, additional confirmation is needed to see if their method performs well even in the actual situation where the model is applied, where low-frequency signals are recorded from the beginning. I recommend publishing this manuscript after responding to the following comments for better Nature communication paper;

(1) The primary objective of this manuscript is to address spiking activities from an extracellular recording obtained in a low temporal resolution potentially insufficient to recognize spiking activities. The authors generated pseudo-data sets from 25-kHz data records through a 200Hz low-pass filter and various downsampling to demonstrate this objective. While their primary focus is restoring spiking activities and spike waveforms, the correlation of 200Hz low-pass data called LFP in the manuscript could be more conceptually ideal. Rather, high-pass filter data (over 200 or 300Hz) can be a better ground to reconstruct spiking activities, which can be a more direct input for the pre-interpolation method and ML approach. The designed approach is oriented to correlate local-field potential to neuronal spiking activity, another important aspect of the brain-machine interface. Please comment on these issues and address the advantages of their LFP-to-spiking reconstruction technique.

(2) In the Authors' detailed analysis, the authors discussed that their ML recognition performance may be derived from the non-perfect characteristics of the Butterworth low-pass filter. As the authors designed the LFP filter with a 200 Hz cutoff frequency (while 100Hz is more general for LFP analysis), the residual high-frequency trait contamination is prone to be expressed in their pseudo-data, which the ML can recognize as spiking pattern recognition. Also, this possibility can be affected by a relative position of spiking activities within the spike-focused window selection and the window size, which may cause application generality, as presented in Figure 6. As pointed out in (1), an idea input for the machine recognition should be the high-pass filtered data directing spiking activities. Please comment on this.

(3) This work aims to advance a software performance of a machine-brain interface, with processing speed being an important consideration alongside accuracy. The proposed algorithm may be time-consuming, consisting of two steps, Fourier pre-interpolation and the Spk-Recon model. And the presented result in Fig 4 shows that the performance varies based on training and evaluation datasets.

And the 8X downsampling comparison with other ML models showed insignificant improvement of Spike-Recon, which the authors further used for the following studies. It would be helpful for readers if the authors compare various ML model characteristics.

(4) Another minor point to consider is the mention of thermal insult in the abstract. It is worth noting that handling a large volume of multichannel electrophysiological data may be more influenced by active electronic components such as head stage amplifiers, which can generate heat and are separate from the recording sites. Therefore, it is unclear whether thermal tissue damage would be a concern related explicitly to high-rate recording hardware design. By addressing the abovementioned concerns, the authors can effectively engage and inform potential readers within the research communities.

(5) Authors need to evaluate the performance of their approach when using neuronal signals recorded low-frequency from the beginning. Their spike reconstruction approach leverages the residual high-frequency components in LPF inputs processed using the Butterworth filter, a non-ideal filter.

(6) Furthermore, the data used in their evaluation is the LPF from neural signals originally recorded with a wide frequency range at high sampling rates. In other words, it can be inferred that the evaluation results for the spike reconstruction shown in this manuscript are guaranteed only when low-pass filtering and down-sampled data are used from neural signals initially recorded with a wide frequency range at high sampling rates. The authors claim that the actual situation in which the model is applied will be recording low-frequency signals at low sampling rates; however, its performance has yet to be demonstrated in the manuscript. Please comment on this issue.

(7) Please show the representative images of cultured neurons grown on MEA1 and MEA2 showing densities and distribution.

(8) Please add the average waveforms of restored spikes detected from in vivo recordings in Figure 7, as the authors argue that the advantage of their approach is the ability to restore entire waveform characteristics.

(10 The spike reconstruction results of the in vivo Hippo dataset (Figures 7f and g) showed a much lower hit rate than the other results. Please show an experiment to investigate if the low spike reconstruction rate was caused by training the Spk-Recon model in different brain regions or by seizure induction.

Reviewer #2 (Remarks to the Author):

This is a very interesting --- and timely --- work, where the authors apply the machine learning technique to detect spikes from extracellular neuronal recording, which has been sub-sampled and low-frequency filtered (to lessen the burden on computing and memory). The detailed utilization of the machine learning algorithm appears credible, and features acceptable accuracy. I would recommend the publication of this manuscript, after following mandatory revisions.

1. I understand the authors would like to emphasize their advantage in terms of being able to handle both spiking and LFPs, and this point is well taken. Now, if we assume only on finding/detecting spiking activities, how does the ML-based method by the authors compare to the traditional/existing methods, in terms of the computing requirement (power consumption, memory usage) and computing time? Probably the authors would have difficult time finding the power consumption, memory (DRAM) usage, and computing time of other methods, but if the authors could provide a perspective on this with at least approximate numbers, that would be helpful for the readers in appreciating the advance put forth by the authors.

2. "Moreover, existing approaches often require custom-designed recording hardware for on-chip signal pre-processing, which also limits universal applicability to state-of-the-art BMI technologies" --- This sentence, which would be important as it seeks to differentiate the authors' work from the traditional approaches, is a bit too difficult to understand. The recording hardware (MEAs) typically and largely shares the same backbone structure (electrodes, followed by integrated amplifiers, and those in an array, with some folks integrating even data converters on chip). In this context, what do authors mean by "require custom-designed recording hardware for on-chip signal pre-processing"? A reasonable explanation would help understand the work better. Also, by "on-chip signal processing," what exact on-chip signal processing are the authors referring to, in terms of what's required by other methods but not by the authors' methods?

3. While the authors focus on extracellular recording data, there has recently emerged a network-wide intracellular recording (Nature Biomedical Engineering 4, 232 (2020)), which has clearer electrode-to-neuron registry, and has a higher signal to noise ratio (much higher) to record not only action potentials or spikes, but also small, sub-threshold synaptic signals (such as post-synaptic potentials of chemical synaptic connections). For a broader perspective of the manuscript, I suggest the authors discuss the applicability and utility of the ML-based strategy in analyzing network-wide intracellular recording, perhaps towards the end of the paper (in Discussion, for example).

4. By "low-sampled", the authors probably refer to "sub-sampled" (i.e., results of sub-sampling)?

Reviewer #3 (Remarks to the Author):

This paper presents a new method for interpreting electrophysiology data collected at low sample rates. Spiking activity detected in extracellular electrophysiology consists largely of data in the range 5-15 kHz, requiring sampling frequencies of 10-30 kHz – perhaps even higher, for some brain regions. As the authors note, sampling at high rates comes at the cost of fewer available recording channels and higher power requirements. Therefore, techniques that allow lower sampling rate are of keen interest, especially for brain-machine interfaces, but also in some research contexts.

The paper explores the possibility of building a machine learning model to reconstruct the high frequency information in electrophysiology data from a low pass filtered (<200 Hz) and downsampled version of that data. They show that their new model, based on a SwinIR transformer architecture, performs this reconstruction very successfully when trained with reasonably similar data for sample rates down to 1/8 of the original 25 kHz. The authors also present valuable insight into how their machine learning model works, showing that it exploits the residual high frequency content that remains after filtering with a 4th order Butterworth filter.

The authors test the model's reconstructions by filtering and downsampling recordings taken at high sample rates, and using the original data as ground truth. Hit rate and similarity of averaged waveforms are two good criteria for evaluating the success of the reconstruction. The different data sets selected for training and testing the model (cultured primary neurons, in vivo recordings in rat cortex, and in vivo recordings in rat hippocampus during seizures) have sufficient variety to demonstrate that the model can be successful when trained on somewhat different data.

Overall this is a good paper on a topic that is important for high-throughput electrophysiology. The text is clear and readable.

Some minor suggestions for improvements:

(1) In addition to hit rate and waveform similarity, false positive rates should be quoted for the reconstructions. Since the training data is enriched for data containing spikes (spike-focused window selection), it's reasonable to be concerned that the reconstruction might create some false spikes.

(2) Because the high frequency information that remains after the 200 Hz low pass filter appears to be important to the final reconstruction (Figure 6 ablation study results) it would be useful to include the post-filtering power spectrum in a more visible plot. The inset in figure 2a is very small and lacks units.

(3) A measurement that would be interesting to add to the ablation results: what happens if the cutoff for the low pass filter is set to a higher frequency? The results show that the lowest useful sample rate is 3125 Hz; at that sample rate, most of the information passed by a 1 kHz cutoff filter would be adequately sampled. The extra high frequency information might significantly enhance the reconstruction. This is of particular interest when considering how well the method could translate to other brain regions (e.g. midbrain) with faster spikes, but might be outside the scope the of the current paper.

(4) It would be helpful to add a bit more interpretation to the functional connectivity assessment in Figure 5b. Is there any measure of how different is "too different" in a BMI context?

Reviewer #1 (Remarks to the Author):

The authors of this manuscript introduced a machine-learning approach to reconstruct high-frequency neuronal spikes using low-sampled low-frequency band signals. Interestingly, their trained model can estimate high-frequency details of spiking activity, such as spike timing, waveform, and network connectivity, even when working with datasets that are down-sampled by a factor of eight. However, their approach and evaluation method may be where reconstruction is guaranteed only when using low-pass filtered and down-sampled data from the neuronal signals initially recorded with a wide frequency range at a high sampling rate. Thus, additional confirmation is needed to see if their method performs well even in the actual situation where the model is applied, where low-frequency signals are recorded from the beginning. I recommend publishing this manuscript after responding to the following comments for better Nature communication paper;

>> We appreciate your overall comment. We provide a point-by-point response to your comments from the next page.

(1) The primary objective of this manuscript is to address spiking activities from an extracellular recording obtained in a low temporal resolution potentially insufficient to recognize spiking activities. The authors generated pseudo-data sets from 25-kHz data records through a 200Hz low-pass filter and various downsampling to demonstrate this objective. While their primary focus is restoring spiking activities and spike waveforms, the correlation of 200 Hz low-pass data called LFP in the manuscript could be more conceptually ideal. Rather, high-pass filter data (over 200 or 300 Hz) can be a better ground to reconstruct spiking activities, which can be a more direct input for the pre-interpolation method and ML approach. The designed approach is oriented to correlate local-field potential to neuronal spiking activity, another important aspect of the brain-machine interface. Please comment on these issues and address the advantages of their LFP-to-spiking reconstruction technique.

>> As we described in the Introduction and Discussion, our Spk-Recon-based spike restoration, which uses low-pass filtered (LPF) signals as an input, can obtain broad bandwidths neuronal signals ranging from LFPs to spikes: low-frequency band LFPs by direct measurement using neural recording hardware and high-frequency band spikes by ML-based reconstruction. This is a significant advantage of our framework compared to methods that directly record high-frequency spikes or restore spikes from high-pass filtered (HPF) signals. With the recorded LFPs, fundamental spectral analyses generally for characterizing brain functions and dynamics can be carried out, as shown in Figs. 1a and 7b. Moreover, using the recorded LFPs and reconstructed spikes together, studies based on LFP-spike correlation[44-47] or spike-triggered LFP averaging[48-50] can also be performed. Additionally, in terms of application to BMI systems, LFP-based BMIs are being developed[51-53] as a way to complement spike-based BMIs that have difficulty in consistent long-term measurement and require a high sampling rate[22-25], and our framework will be compatible with these systems as well. These comments are included in the revised manuscript from Line 57 to Line 63 and from Line 393 to Line 404.

22. Jackson, A. & Hall, T. M. Decoding Local Field Potentials for Neural Interfaces. *IEEE Transactions on Neural Systems and Rehabilitation Engineering* vol. 25 1705–1714 (2017).
23. Milekovic, T. *et al.* Stable long-term BCI-enabled communication in ALS and locked-in syndrome using LFP signals. *J. Neurophysiol.* **120**, 343–360 (2018).
24. Heldman, D. A. & Moran, D. W. Local field potentials for BCI control. in *Handbook of Clinical Neurology* vol. 168 279–288 (2020).
25. Nazari, M. R., Nasrabadi, A. M. & Daliri, M. R. Single-Trial Decoding of Motion Direction during Visual Attention from Local Field Potential Signals. *IEEE Access* **9**, 66450–66461 (2021).
44. Martin, K. A. C. & Schröder, S. Phase locking of multiple single neurons to the local field potential in cat V1. *J. Neurosci.* **36**, 2494–2502 (2016).
45. Telenczuk, B. *et al.* Local field potentials primarily reflect inhibitory neuron activity in human and monkey cortex. *Sci. Rep.* **7**, 1–10 (2017).
46. Parto Dezfouli, M., Zarei, M., Jahed, M. & Daliri, M. R. Stimulus-specific adaptation decreases the coupling of spikes to LFP phase. *Front. Neural Circuits* **13**, 44 (2019).
47. Xu, W., De Carvalho, F. & Jackson, A. Sequential neural activity in primary motor cortex during sleep. *J. Neurosci.* **39**, 3698–3712 (2019).
48. Nauhaus, I., Busse, L., Carandini, M. & Ringach, D. L. Stimulus contrast modulates functional connectivity in visual cortex. *Nat. Neurosci. 2008 121* **12**, 70–76 (2008).
49. Ray, S. & Maunse ll, J. H. R. Network rhythms influence the relationship between spike- triggered local field potential and functional connectivity. *J. Neurosci.* **31**, 12674–12682 (2011).
50. Swindale, N. V. & Spacek, M. A. Visual cortex neurons phase-lock selectively to subsets of LFP oscillations. *J. Neurophysiol.* **121**, 2364–2378 (2019).
51. Perel, S. *et al.* Single-unit activity, threshold crossings, and local field potentials in motor cortex differentially encode reach kinematics. *J. Neurophysiol.* **114**, 1500–1512 (2015).
52. Gilja, V. *et al.* Clinical translation of a high-performance neural prosthesis. *Nat. Med.* **21**, 1142–1145 (2015).
53. Peles, O., Werner-Reiss, U., Bergman, H., Israel, Z. & Vaadia, E. Phase-Specific Microstimulation Differentially Modulates Beta Oscillations and Affects Behavior. *Cell Rep.* **30**, 2555-2566.e3 (2020).

(2) In the Authors' detailed analysis, the authors discussed that their ML recognition performance may be derived from the non-perfect characteristics of the Butterworth low-pass filter. As the authors designed the LFP filter with a 200 Hz cutoff frequency (while 100 Hz is more general for LFP analysis), the residual high-frequency trait contamination is prone to be expressed in their pseudo-data, which the ML can recognize as spiking pattern recognition. Also, this possibility can be affected by a relative position of spiking activities within the spike-focused window selection and the window size, which may cause application generality, as presented in Figure 6. As pointed out in (1), an idea input for the machine recognition should be the high-pass filtered data directing spiking activities. Please comment on this.

>> As the reviewer pointed out, we attempted to train and evaluate our Spk-Recon model using subsampled HPF signals with a factor of 8 as an input instead of LPF signals. As shown in the restored traces below, the model trained using HPF input and HPF GT signals can also reconstruct the signal similar to the corresponding GT signal, but with slightly degraded performance: lower mean hit rate (HPF input: 0.75 ± 0.14 vs. LPF input: 0.78 ± 0.14) and higher mean NRMSE (HPF input: 0.09 ± 0.01 vs. LPF input: 0.07 ± 0.01) (mean ± SD, n = 13 electrodes). Using the HPF input signal can be an alternative. However, to take advantage of what was described in the answer (1), we adopted the LPF signal as the input for spike reconstruction.

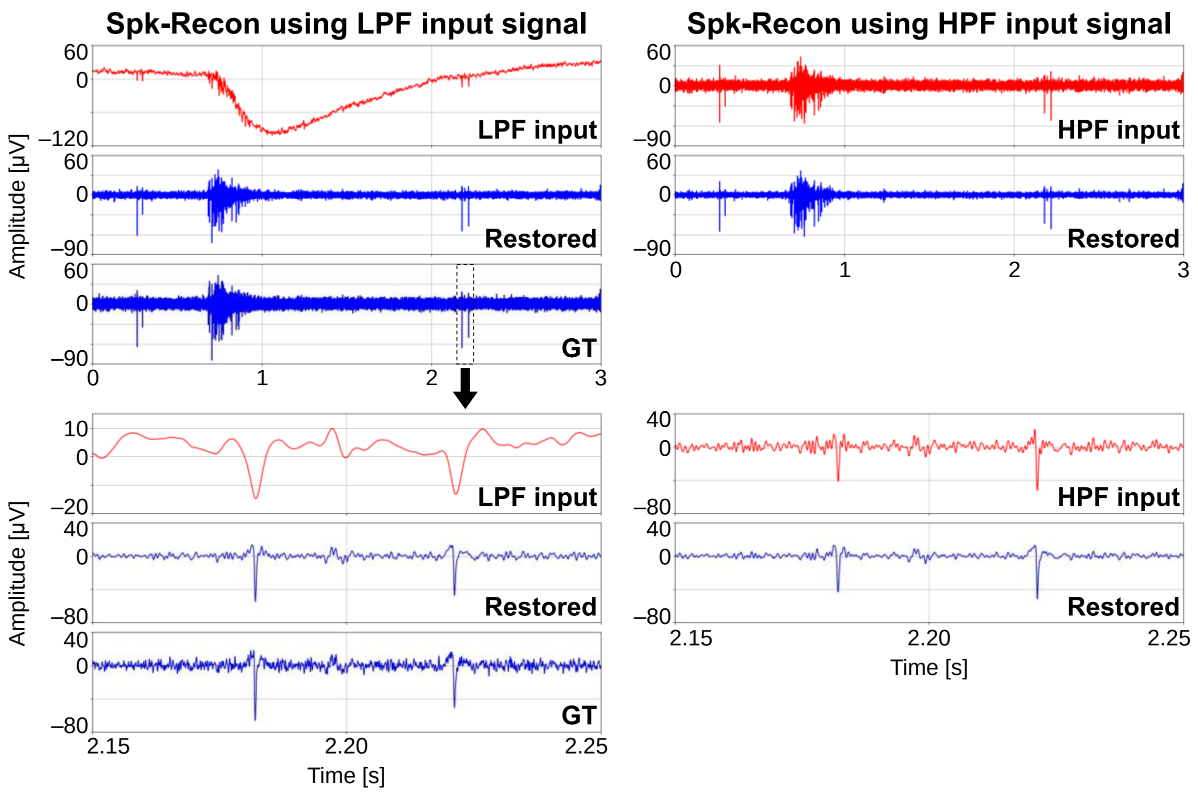

(3) This work aims to advance a software performance of a machine-brain interface, with processing speed being an important consideration alongside accuracy. The proposed algorithm may be time-consuming, consisting of two steps, Fourier pre-interpolation and the Spk-Recon model. And the presented result in Fig 4 shows that the performance varies based on training and evaluation datasets. And the 8X downsampling comparison with other ML models showed insignificant improvement of Spike-Recon, which the authors further used for the following studies. It would be helpful for readers if the authors compare various ML model characteristics.

>> For real-time applicability, further research such as a lightweight model (e.g., using a lightweight deep neural network such as MobileNets [1]) would be necessary. Nevertheless, in running our algorithm consisting of two steps (pre-interpolation using the Fourier method and signal reconstruction using the transformer-based model), the computation time for the pre-interpolation was much shorter than that for signal reconstruction from the model (pre-interpolation: 0.09 ms vs. signal reconstruction: 36.29 ms; mean computation time over 300 repetitions for a single data sequence with a downsampling factor of 8), having little impact on total running time. In response to the reviewer's comment, we have added the following sentence in the main text:

"(Line 158)… In implementing our algorithm, the computation time for this pre-interpolation was much shorter than that for signal reconstruction from the Spk-Recon model (pre-interpolation: 0.09 ms vs. signal reconstruction: 36.29 ms; mean computation time over 300 repetitions for a single data sequence with a downsampling factor of 8), having little impact on total running time."

[1] Howard, Andrew G., et al. "Mobilenets: Efficient convolutional neural networks for mobile vision applications." *arXiv preprint arXiv:1704.04861* (2017).

>> In the restoration results of the downsampling factor of 8 (Fig. 4b and c), the hit rates of the Spk-Recon were higher than those of the other models but without statistical significance. However, in all factors, the Spk-Recon showed superior performance in reconstructing spike waveforms compared to the other models, as indicated by significantly lower NRMSE values (Fig. 4d and e). We have added the following sentences about comparison results in the main text:

"(Line 205)… Taken together, the results demonstrated that the Spk-Recon achieved the best performance in reconstructing the accurate spike timings and waveforms on both MEA datasets. In particular, for the downsampling factor of 1, 16, and 25, the hit rates of Spk-Recon were significantly higher than those of CNN-based TCN and EDSR or transformer-based SwinIR. Moreover, the Spk-Recon showed significant improvement in reconstructing spike waveforms compared to the other models across all the downsampling factors."

(4) Another minor point to consider is the mention of thermal insult in the abstract. It is worth noting that handling a large volume of multichannel electrophysiological data may be more influenced by active electronic components such as head stage amplifiers, which can generate heat and are separate from the recording sites. Therefore, it is unclear whether thermal tissue damage would be a concern related explicitly to high-rate recording hardware design. By addressing the abovementioned concerns, the authors can effectively engage and inform potential readers within the research communities.

>> In the case of a CMOS-based active neural probe with multiplexing electronics integrated into the probe shank or a wireless neural sensor that is fully implanted into the brain, the heat dissipation due to high power consumption can cause thermal damage to the biological tissues with which the neural probe or sensor is in contact. In response to the reviewer's comment, we have added the following sentence in the main text:

"(Line 47)… Moreover, higher numbers of electrodes require larger storage memory and induce higher power consumption in recording and wireless communication, resulting in significant heat dissipation. Such heating should be avoided in implantable BMIs because it can cause thermal damage to surrounding biological tissues, especially for CMOS-based active neural probes that multiplexing electronics are integrated into the probe shank[5] or for wireless neural sensors that are fully implanted[10–12]."

5. Hong, G. & Lieber, C. M. Novel electrode technologies for neural recordings. *Nature Reviews Neuroscience* vol. 20 330–345 (2019).
10. Biederman, W. *et al.* A fully-integrated, miniaturized (0.125 mm2) 10.5 µw wireless neural sensor. *IEEE J. Solid-State Circuits* **48**, 960–970 (2013).
11. Seo, D., Carmena, J. M., Rabaey, J. M., Maharbiz, M. M. & Alon, E. Model validation of untethered, ultrasonic neural dust motes for cortical recording. *J. Neurosci. Methods* **244**, 114–122 (2015).
12. Lee, J. *et al.* Neural recording and stimulation using wireless networks of microimplants. *Nat. Electron.* **4**, 604–614 (2021).

(5) Authors need to evaluate the performance of their approach when using neuronal signals recorded low-frequency from the beginning. Their spike reconstruction approach leverages the residual high-frequency components in LPF inputs processed using the Butterworth filter, a non-ideal filter.
(6) Furthermore, the data used in their evaluation is the LPF from neural signals originally recorded with a wide frequency range at high sampling rates. In other words, it can be inferred that the evaluation results for the spike reconstruction shown in this manuscript are guaranteed only when low-pass filtering and down-sampled data are used from neural signals initially recorded with a wide frequency range at high sampling rates. The authors claim that the actual situation in which the model is applied will be recording low-frequency signals at low sampling rates; however, its performance has yet to be demonstrated in the manuscript. Please comment on this issue.

>> As the review pointed out, we attempted to reconstruct the high-frequency signal with a downsampling factor of 8 using neuronal signals recorded from in vivo mouse hippocampus with low-frequency bands (< 200 Hz) from the beginning. The restored signal (red trace) shows that the Spk-Recon model reconstructed the voltage fluctuations, including spiking events. However, we could not confirm whether the spikes detected from the restored signal were correct or not because the corresponding high-frequency signal could not be recorded simultaneously while recording the low-frequency signal. Please find the graphs in the next page.

As mentioned in the main text, pairs of low-frequency input and high-frequency GT signals are needed for model training via supervised learning, where the model is trained on a labeled dataset. Moreover, to evaluate the performance of the trained model to determine whether it reconstructs spike timings and waveforms correctly, it is necessary to compare the reconstructed signal to the corresponding high-frequency GT signal. Unfortunately, however, in the commercial systems we used (Multi Channel Systems, NeuroNexus, and Intan Technologies), there was no way to acquire the high-frequency GT signal simultaneously while recording the low-frequency signal from the beginning. Due to this experimental limitation, in this study, we recorded wide-frequency signals, separated them into LPF and HPF signals through software filtering, and used them for model training and evaluation. To demonstrate the spike reconstruction from neuronal signals recorded with low-frequency bands, further study is needed on developing neural recording hardware that can record low-frequency signals while acquiring high-frequency signals originating from the same neural source. We hope you understand this practical limitation and look forward to reporting such a demonstration soon.

Instead of the quantitative performance measures such as hit rate or waveform NRMSE, we tried to compare the signal with the HPF signal (blue trace) that was recorded with a wide frequency range after low-frequency signal recording. As shown in signal traces and average spike waveforms (red and blue for the restored signal and the measured HPF signal, respectively), the restored signal had a smaller amplitude with a wider spike waveform. We believe this poorer performance is because our ML model was trained on the dataset using a different signal filter than the one we used in this study. As stated above, we expect better model training for improved signal restoration and quantitative performance evaluation based on one-to-one comparison with GT spikes can be achieved through further study on developing neural recording hardware to acquire low- and high-frequency signals separately but simultaneously. Nevertheless, since many neuroscientists and neural engineers are collecting and analyzing neuronal signals in the desired frequency range through the software filtering provided by commercial systems such as the above, our proposed algorithm would still be highly utilized.

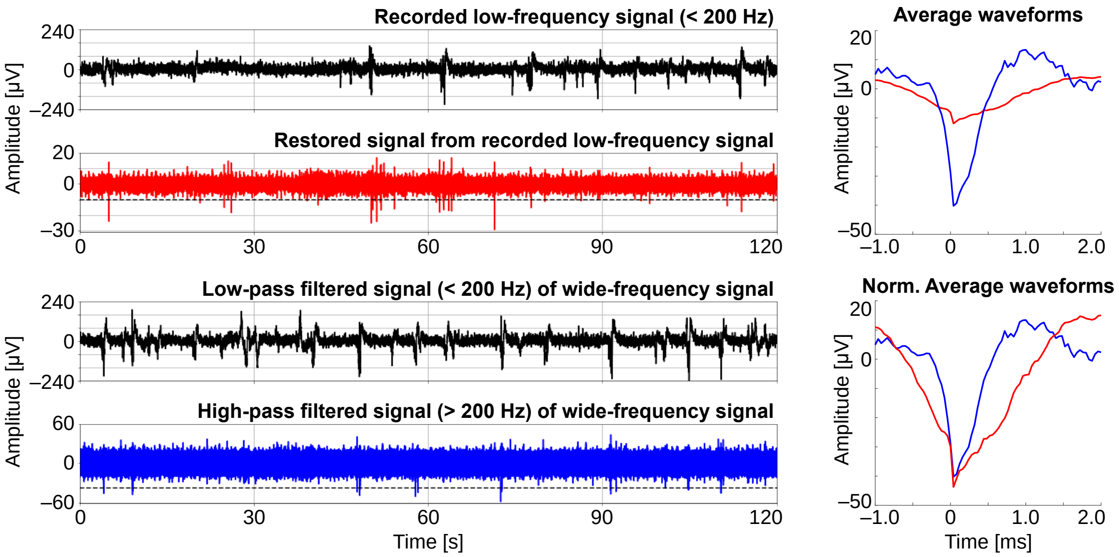

Recorded low-frequency signal (< 200 Hz)
Restored signal from recorded low-frequency signal
Low-pass filtered signal (< 200 Hz) of wide-frequency signal
High-pass filtered signal (> 200 Hz) of wide-frequency signal
Average waveforms
Norm. Average waveforms
Amplitude [μV]
Time [s]
Time [ms]
240
0
−240
20
−30
60
−60
−50
30
90
120
−1.0
1.0
2.0

(7) Please show the representative images of cultured neurons grown on MEA1 and MEA2 showing densities and distribution.

>> In response to the reviewer’s comment, we have added phase-contrast images of cultured neuronal networks on MEA1 and MEA2 in Supplementary information (Supplementary Fig. 1).

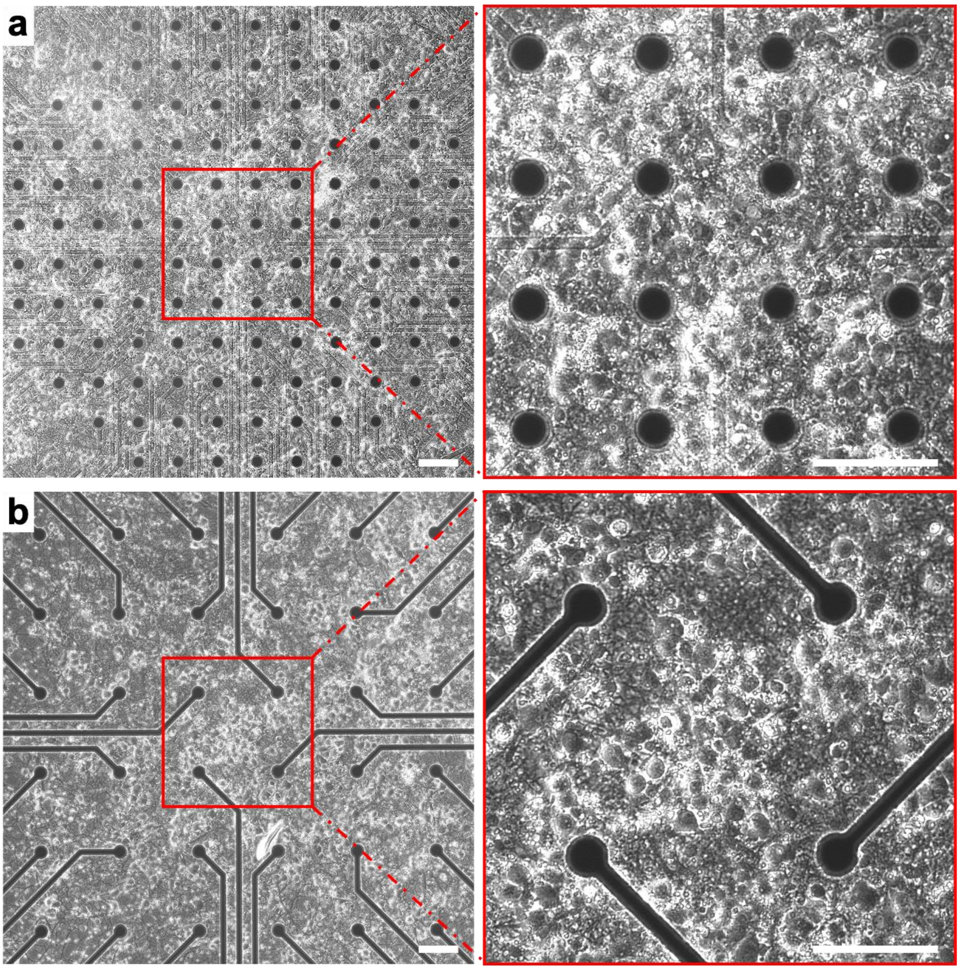

Supplementary Fig. 1 | In vitro neuronal networks cultured on MEAs for two weeks. a Phase-contrast image of the cultured neuronal network on the MEA1. **b** Phase-contrast image of the cultured neuronal network on the MEA2. Scale bar: 100 μm.

(8) Please add the average waveforms of restored spikes detected from in vivo recordings in Figure 7, as the authors argue that the advantage of their approach is the ability to restore entire waveform characteristics.

>> In response to the reviewer's comment, we have added average waveforms of restored spikes from in vivo recordings in Fig. 7.

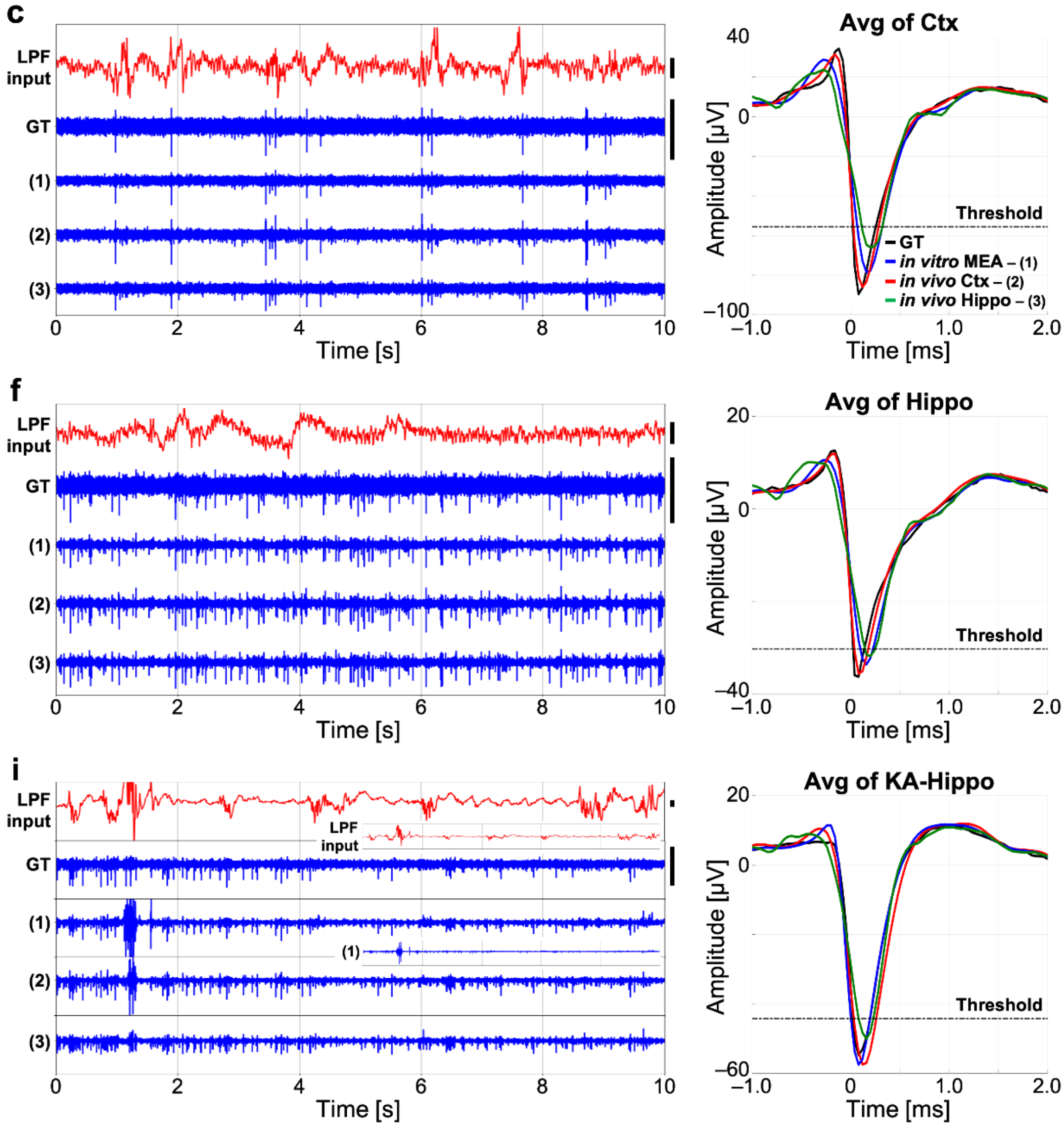

Fig. 7. Signal restoration on in vivo datasets with the downsampling factor of 8. (c) Reconstructed Ctx signals and their average spike waveforms using the Spk-Recon models trained on three different datasets (Trained on (1) in vitro MEA, (2) in vivo Ctx, or (3) in vivo Hippo datasets). Except for the LPF input signals, all other signals are plotted using the same scales. Scale bar: 200 µV. (f) Reconstructed Hippo signals and their average spike waveforms. Except for the LPF input signals, all other signals are plotted using the same scales. Scale bar: 100 µV. (i) Reconstructed KA-Hippo signals of the KA-treated mouse and their average spike waveforms. Except for the LPF input signals, all other signals are plotted using the same scales. Scale bar: 200 µV. Insets display the same traces of the LPF input and the restored signal (1) on different y-axis scales (y-axis of the LPF input: –2 ~ 2 mV; y-axis of the restored signal (1): –500 ~ 400 µV).

(9) The spike reconstruction results of the in vivo Hippo dataset (Figures 7f and g) showed a much lower hit rate than the other results. Please show an experiment to investigate if the low spike reconstruction rate was caused by training the Spk-Recon model in different brain regions or by seizure induction.

>> In response to the reviewer's comment, we have conducted an additional experiment on signal restoration on an in vivo dataset recorded from the mouse hippocampus without seizure induction by KA treatment. We have reorganized Fig. 7 and revised the related paragraph from Line 321 to clarify these results.

We trained the Spk-Recon models with three different training datasets ((1) in vitro MEA, (2) in vivo Ctx, and (3) in vivo Hippo datasets (new dataset without KA treatment)). We evaluated these trained models on the three in vivo test datasets: Ctx, Hippo (new dataset without KA treatment), and KA-Hippo (dataset with KA treatment, previously called Hippo). The results of the signal restoration on the three in vivo test datasets are shown in Fig. 7.

For the Ctx test dataset, three models trained on (1) in vitro MEA, (2) in vivo Ctx, and (3) in vivo Hippo training datasets reconstructed high-frequency signals with high hit rates (mean values ranging from 0.87 to 0.97).

For both the Hippo and KA-Hippo test datasets, the hit rate using the model trained (1) in vitro MEA dataset was low (0.54 and 0.55). Those of the other models trained (2) in vivo Ctx and (3) in vivo Hippo were high (mean values ranging from 0.78 to 0.81), which were comparable to the reconstruction performance of the in vitro test datasets (Fig. 4b, c).

This implies that the model trained under the in vivo condition performs better at the signal reconstruction of in vivo recordings; that is, the low reconstruction performance in the Hippo and KA-Hippo test datasets was caused by training the model under an in vitro condition, not by different brain regions or seizure induction.

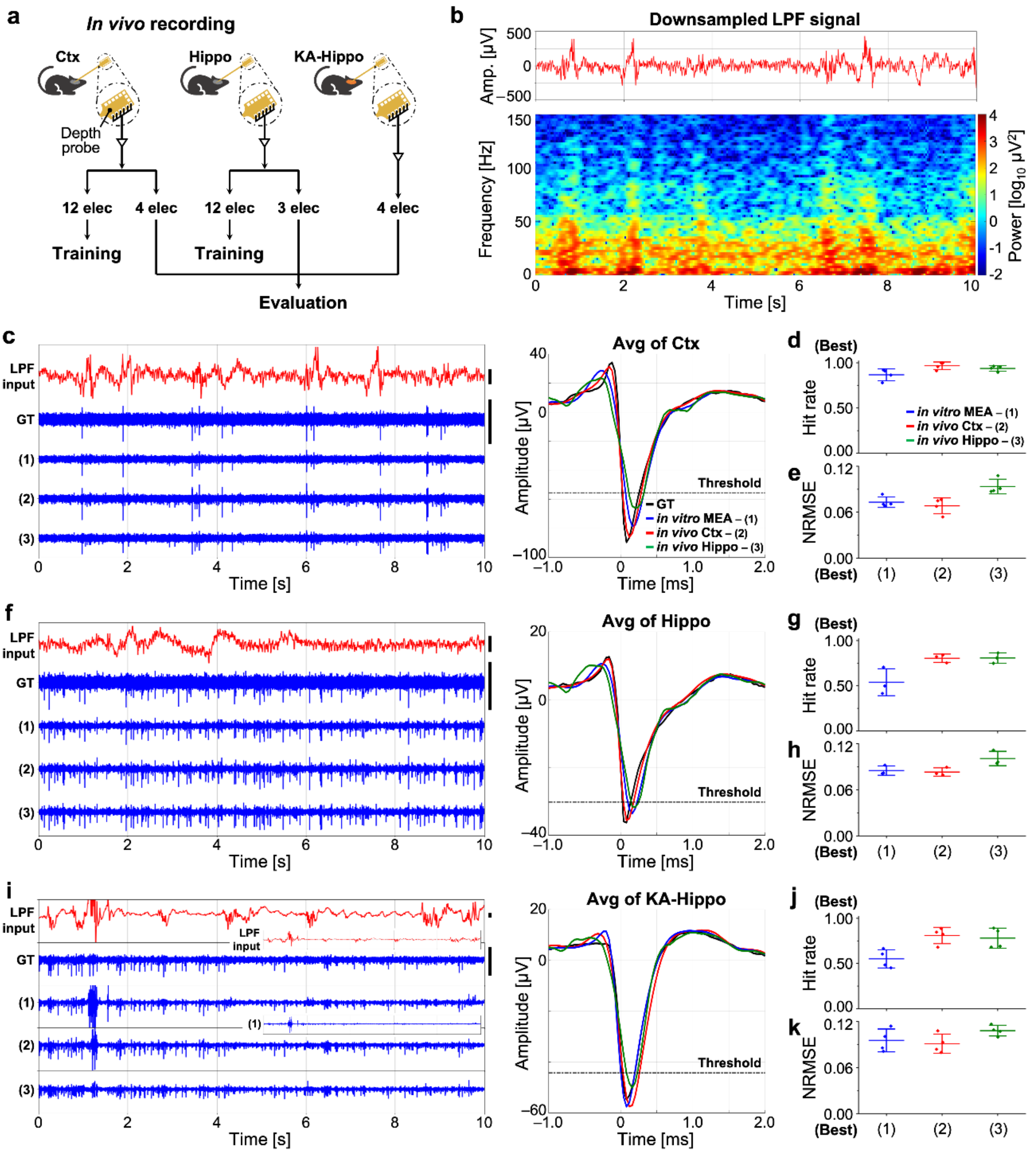

Fig. 7. Signal restoration on in vivo datasets with the downsampling factor of 8. (a) Signal processing of cortex (Ctx), hippocampus (Hippo), and KA-treated mouse hippocampus (KA-Hippo) datasets for model training and evaluation. (b) Raw trace and spectrogram of the downsampled LPF signal obtained from the Ctx before the interpolation process. (c) Reconstructed Ctx signals and their average spike waveforms using the Spk-Recon models trained on three different datasets (Trained on (1) in vitro MEA, (2) in vivo Ctx, or (3) in vivo Hippo datasets). Except for the LPF input signals, all other signals are plotted using the same scales. Scale bar: 200 µV. (d) Hit rate of spike detection and (e) NRMSE between the restored and actual waveforms of the Ctx dataset. n = 4 electrodes. Note that a hit rate of 1 means that all the GT spikes are correctly reconstructed in timing, and an NRMSE of 0 means that spike waveforms are perfectly restored. (f) Reconstructed Hippo signals and their average spike waveforms. Except for the LPF input signals, all other signals are plotted using the same scales. Scale bar: 100 µV. (g) Hit rate of spike detection and (h) NRMSE between the restored and actual waveforms of the Hippo dataset. n = 3 electrodes. (i) Reconstructed KA-Hippo signals of the KA-treated mouse and their average spike waveforms. Except for the LPF input signals, all other signals are plotted using the same scales. Scale bar: 200 µV. Insets display the same traces of the LPF input and the restored signal (1) on different y-axis scales (y-axis of the LPF input: –2 ~ 2 mV; y-axis of the restored signal (1): –500 ~ 400 µV). (j) Hit rate of spike detection and (k) NRMSE between the restored and actual waveforms of the KA-Hippo dataset. n = 4 electrodes. Data are presented as mean ± SD. Source data are provided as a Source Data file.

Reviewer #2 (Remarks to the Author):

This is a very interesting --- and timely --- work, where the authors apply the machine learning technique to detect spikes from extracellular neuronal recording, which has been sub-sampled and low-frequency filtered (to lessen the burden on computing and memory). The detailed utilization of the machine learning algorithm appears credible, and features acceptable accuracy. I would recommend the publication of this manuscript, after following mandatory revisions.

1. I understand the authors would like to emphasize their advantage in terms of being able to handle both spiking and LFPs, and this point is well taken. Now, if we assume only on finding/detecting spiking activities, how does the ML-based method by the authors compare to the traditional/existing methods, in terms of the computing requirement (power consumption, memory usage) and computing time? Probably the authors would have difficult time finding the power consumption, memory (DRAM) usage, and computing time of other methods, but if the authors could provide a perspective on this with at least approximate numbers, that would be helpful for the readers in appreciating the advance put forth by the authors.

>> Thank you very much for your positive overall comments.

While we want to compare the computing performances across different methodologies, it is very difficult. We hope you understand that we cannot answer because it is hard to find an appropriate reference, as the reviewer mentioned. Also, it is outside the exact scope of our current work. The present work is to develop an ML-based algorithm for reconstructing high-frequency band signals with high temporal resolution, which include spike information, from low-frequency band signals with a low temporal resolution. In the near future, we would work on optimizing the ML algorithm for lower computation load and even perhaps running on more portable ML hardware. During that effort, we may be able to evaluate the computing power requirement more accurately.

For spike identification from the reconstructed high-frequency band signals to evaluate signal reconstruction performance, we used the threshold setting method, which is one of the traditional ones, instead of detecting spikes directly via the ML model.

2. "Moreover, existing approaches often require custom-designed recording hardware for on-chip signal pre-processing, which also limits universal applicability to state-of-the-art BMI technologies" --- This sentence, which would be important as it seeks to differentiate the authors' work from the traditional approaches, is a bit too difficult to understand. The recording hardware (MEAs) typically and largely shares the same backbone structure (electrodes, followed by integrated amplifiers, and those in an array, with some folks integrating even data converters on chip). In this context, what do authors mean by "require custom-designed recording hardware for on-chip signal pre-processing"? A reasonable explanation would help understand the work better. Also, by "on-chip signal processing," what exact on-chip signal processing are the authors referring to, in terms of what's required by other methods but not by the authors' methods?

>> The 'On-chip signal pre-processing' we described refers to signal processing before the neuronal signal, measured from electrodes, is transmitted to a computer. As the reviewer mentioned, neuronal signals are commonly passed through electrodes, amplifiers, filters, ADCs, and computers. However, in order to reduce the volume of data in advance before signal transmission using a specific algorithm, as in the existing studies cited as examples, additional circuitry is required for neural recording hardware. For example, in the case of compressed sensing[15] or on-chip compression[18,19], encoders are essential to implement individual algorithms. Additionally, in the case of downgrading signal qualities[17] and on-chip spike detection[20], circuitries for detecting spikes by identifying threshold-crossing events or characterizing spike waveforms and for converting spikes into binary form are required. On the other hand, our method of lowering the frequency band and sampling rate of the acquired signals is differentiated in that it can be fully implemented within the basic backbone structure. In response to the reviewer's comment, we have revised that sentence as follows:

"(Line 63)… Moreover, existing approaches often require custom-designed recording hardware for on-chip signal pre-processing before data transmission, such as encoders for implementing individual algorithms or additional circuitries for detecting spikes by identifying threshold-crossing events or characterizing spike waveforms and for converting spikes into binary form. This also limits universal applicability to state-of-the-art BMI technologies."

15. Sun, B. & Zhao, W. Compressed Sensing of Extracellular Neurophysiology Signals: A Review. *Frontiers in Neuroscience* vol. 15 948 (2021).
17. Even-Chen, N. *et al.* Power-saving design opportunities for wireless intracortical brain–computer interfaces. *Nat. Biomed. Eng.* **4**, 984–996 (2020).
18. Wu, T., Zhao, W., Keefer, E. & Yang, Z. Deep compressive autoencoder for action potential compression in large-scale neural recording. *J. Neural Eng.* **15**, 066019 (2018).
19. Muratore, D. G. *et al.* A data-compressive wired-or readout for massively parallel neural recording. in *Proceedings - IEEE International Symposium on Circuits and Systems* vols 2019-May (Institute of Electrical and Electronics Engineers Inc., 2019).
20. Yoon, D. Y. *et al.* A 1024-Channel Simultaneous Recording Neural SoC with Stimulation and Real-Time Spike Detection. in *IEEE Symposium on VLSI Circuits, Digest of Technical Papers* vols 2021-June (2021).

3. While the authors focus on extracellular recording data, there has recently emerged a network-wide intracellular recording (Nature Biomedical Engineering 4, 232 (2020)), which has clearer electrode-to-neuron registry, and has a higher signal to noise ratio (much higher) to record not only action potentials or spikes, but also small, sub-threshold synaptic signals (such as post-synaptic potentials of chemical synaptic connections). For a broader perspective of the manuscript, I suggest the authors discuss the applicability and utility of the ML-based strategy in analyzing network-wide intracellular recording, perhaps towards the end of the paper (in Discussion, for example).

>> Thank you very much for your suggestion. Though we were not able to attempt to correlate the intracellular signals, we also thought that it would be an additional area that our algorithm could be applied to. In response to the reviewer's comment, we have cited the article and added the following sentence in the Discussion:

"(Line 421)… In addition, by utilizing the latest MEA technologies that enable network-wide intracellular recording[54], the reconstruction of intracellular neuronal signals, such as action potentials or subthreshold synaptic signals, from extracellular recordings could also be achieved."

54. Abbott, J. et al. A nanoelectrode array for obtaining intracellular recordings from thousands of connected neurons. *Nat. Biomed. Eng.* **4**, 232–241 (2020).

4. By "low-sampled", the authors probably refer to "sub-sampled" (i.e., results of sub-sampling)?

>> In response to the reviewer's comment, we have replaced the word "low-sampled" with "subsampled".

Reviewer #3 (Remarks to the Author):

This paper presents a new method for interpreting electrophysiology data collected at low sample rates. Spiking activity detected in extracellular electrophysiology consists largely of data in the range 5-15 kHz, requiring sampling frequencies of 10-30 kHz – perhaps even higher, for some brain regions. As the authors note, sampling at high rates comes at the cost of fewer available recording channels and higher power requirements. Therefore, techniques that allow lower sampling rate are of keen interest, especially for brain-machine interfaces, but also in some research contexts.

The paper explores the possibility of building a machine learning model to reconstruct the high frequency information in electrophysiology data from a low pass filtered (<200 Hz) and downsampled version of that data. They show that their new model, based on a SwinIR transformer architecture, performs this reconstruction very successfully when trained with reasonably similar data for sample rates down to 1/8 of the original 25 kHz. The authors also present valuable insight into how their machine learning model works, showing that it exploits the residual high frequency content that remains after filtering with a 4th order Butterworth filter.

The authors test the model's reconstructions by filtering and downsampling recordings taken at high sample rates, and using the original data as ground truth. Hit rate and similarity of averaged waveforms are two good criteria for evaluating the success of the reconstruction. The different data sets selected for training and testing the model (cultured primary neurons, in vivo recordings in rat cortex, and in vivo recordings in rat hippocampus during seizures) have sufficient variety to demonstrate that the model can be successful when trained on somewhat different data.

Overall this is a good paper on a topic that is important for high-throughput electrophysiology. The text is clear and readable.

>> We appreciate your overall comment. We provide our responses to your questions on the next page.

Some minor suggestions for improvements:
(1) In addition to hit rate and waveform similarity, false positive rates should be quoted for the reconstructions. Since the training data is enriched for data containing spikes (spike-focused window selection), it's reasonable to be concerned that the reconstruction might create some false spikes.

>> In response to the reviewer's comment, we have appended a table of precision, which is defined as the ratio of the number of correctly restored spikes (true positive) to that of spikes detected in the reconstructed signal (true positive + false positive), in Supplementary information (Supplementary Table 1) and added the following sentences in the main text:

"(Line 201)… Additionally, we figured out the precision of spike detection, which is defined as the ratio of the number of correctly restored spikes (true positive) to that of spikes detected in the reconstructed signal (true positive + false positive). The mean precision values of the Spk-Recon were high in all conditions, ranging from 0.89 to 0.97, implying that most detected spikes occurred at correct timings."

Supplementary Table 1 | Precision of spike detection on two MEAs with different models and downsampling factors. The precision is defined as the ratio of the number of correctly restored spikes to that of spikes detected in the reconstructed signal. Data are presented as mean ± SD.

**Training dataset:** MEA1 (n = 100)		**Downsampling factor** ***M***			
**Test dataset**	**Model**	1	8	16	25
MEA1 (n = 13)	TCN	0.91 ± 0.07	0.91 ± 0.07	0.88 ± 0.06	0.91 ± 0.08
	EDSR	0.98 ± 0.01	0.96 ± 0.04	0.94 ± 0.07	0.82 ± 0.15
	SwinIR	0.85 ± 0.07	0.96 ± 0.04	0.94 ± 0.07	0.77 ± 0.15
	Spk-Recon	0.93 ± 0.04	0.95 ± 0.03	0.89 ± 0.07	0.91 ± 0.08
MEA2 (n = 16)	TCN	0.95 ± 0.05	0.94 ± 0.06	0.91 ± 0.06	0.94 ± 0.06
	EDSR	0.99 ± 0.01	0.98 ± 0.02	0.97 ± 0.04	0.91 ± 0.09
	SwinIR	0.90 ± 0.07	0.98 ± 0.02	0.98 ± 0.03	0.85 ± 0.12
	Spk-Recon	0.96 ± 0.03	0.97 ± 0.02	0.92 ± 0.07	0.92 ± 0.10

(2) Because the high frequency information that remains after the 200 Hz low pass filter appears to be important to the final reconstruction (Figure 6 ablation study results) it would be useful to include the post-filtering power spectrum in a more visible plot. The inset in figure 2a is very small and lacks units.

>> In response to the reviewer's comment, we have added power spectral density graphs of the raw signal before filtering and the low-pass filtered (LPF) signals after filtering in Supplementary information (Supplementary Fig. 2).

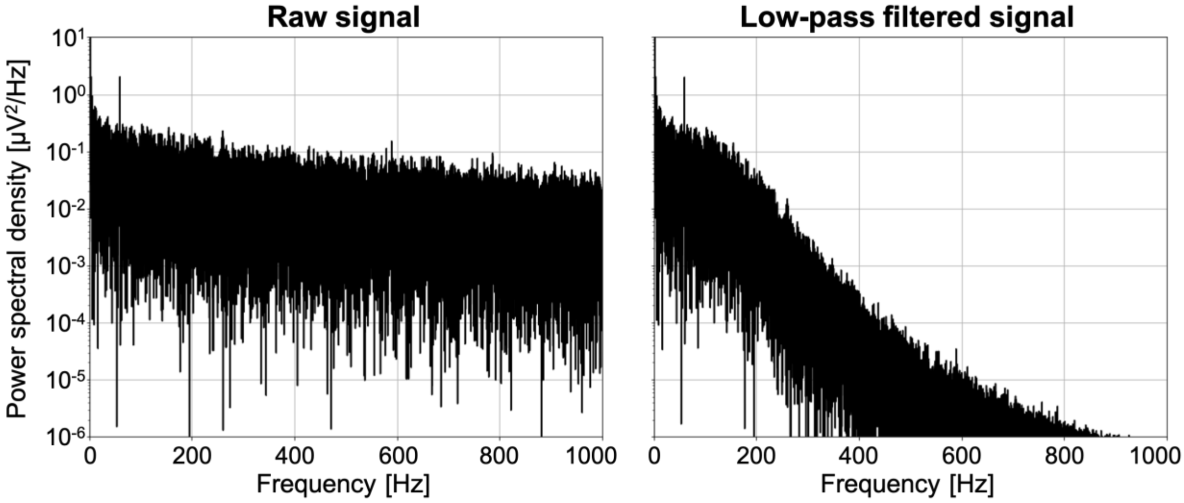

Supplementary Fig. 2 | Power spectral densities of raw and low-pass filtered signals.

(3) A measurement that would be interesting to add to the ablation results: what happens if the cutoff for the low pass filter is set to a higher frequency? The results show that the lowest useful sample rate is 3125 Hz; at that sample rate, most of the information passed by a 1 kHz cutoff filter would be adequately sampled. The extra high frequency information might significantly enhance the reconstruction. This is of particular interest when considering how well the method could translate to other brain regions (e.g. midbrain) with faster spikes, but might be outside the scope the of the current paper.

>> As the reviewer pointed out, we attempted to train and evaluate our Spk-Recon model using LPF signals with a cutoff frequency of 1 kHz instead of LPF input signals with a cutoff frequency of 200 Hz for a downsampling factor of 8 (sampling rate of 3125 Hz). When using LPF signals filtered with the 1 kHz cutoff frequency as an input, the trained model restored the signal similar to the corresponding HPF GT signal with slightly enhanced performance: higher mean hit rate (1 kHz cutoff: 0.82 ± 0.10 vs. 200 Hz cutoff: 0.78 ± 0.14) and identical mean NRMSE (0.07 ± 0.01) (mean ± SD, n = 13 electrodes). Please see the example waveform below.

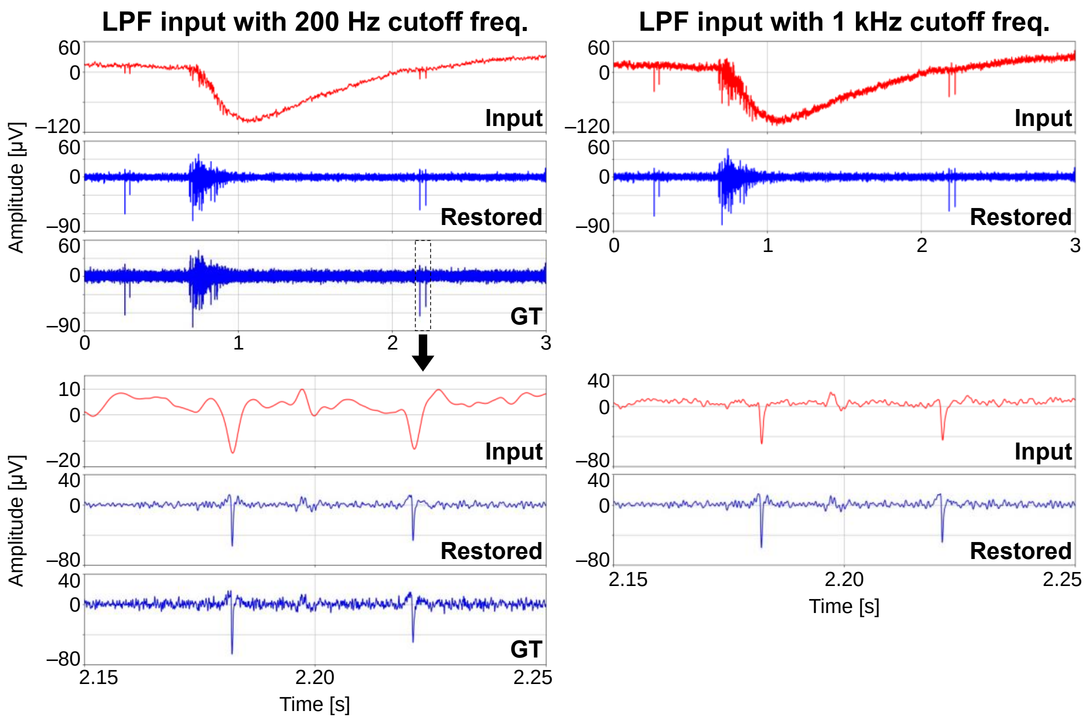

In this study, setting the cutoff frequency at a high frequency of about 1 kHz is impossible because the downsampling factors of 16 and 25 were also used, but it can be one of the strategies to achieve improved performance if a downsampling factor of 8 or smaller is used in the future.

(4) It would be helpful to add a bit more interpretation to the functional connectivity assessment in Figure 5b. Is there any measure of how different is "too different" in a BMI context?

>> In general, functional connectivity is assessed using measures such as a Pearson correlation coefficient, which is the normalized measure used in this study, and significant changes in the value itself are analyzed to identify differences in brain states between normal and pathological conditions or to clarify the effect of external stimulation and manipulation on network connectivity. In response to the reviewer's comment, we have added the result of the statistical comparison with the coefficients of the GT and the following sentences in the main text:

"(Line 238)… Despite some missing spikes and slightly shifted timestamps, the correlation matrices for the downsampling factors up to 8 were similar to that of the GT without significant differences in the coefficients (p = 0.7243, 0.1542, 0.0131 and < 0.0001 for the factor 1, 8, 16, and 25, respectively; Two-tailed two-sample t-test for the coefficients compared with those of the GT), indicating that the spatial network connectivity was reasonably well re-established. This accurate restoration ability is essential for detecting significant changes in functional connectivity to identify differences in brain states between normal and pathological conditions[33,34] or to clarify the effect of external stimulation and manipulation on network connectivity[35,36]."

33. Zhang, C. *et al.* Characteristics of resting-state functional connectivity in intractable unilateral temporal lobe epilepsy patients with impaired executive control function. *Front. Hum. Neurosci.* **11**, 288310 (2017).
34. Vallesi, V. *et al.* Functional connectivity and amplitude of low-frequency fluctuations changes in people with complete subacute and chronic spinal cord injury. *Sci. Rep.* **12**, 1–8 (2022).
35. Bai, Y. *et al.* Deep Brain Stimulation Modulates Multiple Abnormal Resting-State Network Connectivity in Patients With Parkinson's Disease. *Front. Aging Neurosci.* **14**, 794987 (2022).
36. Gaebler, A. J. *et al.* Functional connectivity signatures of NMDAR dysfunction in schizophrenia—integrating findings from imaging genetics and pharmaco-fMRI. *Transl. Psychiatry* **13**, 1–12 (2023).

REVIEWERS' COMMENTS

Reviewer #1 (Remarks to the Author):

This reviewer is satisfied with all responses to questions and happy with the revised manuscript.

Reviewer #2 (Remarks to the Author):

The authors have largely addressed my prior comments. They could not address comment 1, but I predicted it to be a difficult task. I would like to recommend publication.

Reviewer #3 (Remarks to the Author):

Final review after revisions:

I believe the authors have addressed both my comments and those of the other two reviewers. The result is definitely of significant interest—reading the questions of the other reviewers gave me some more insight into the importance of such techniques in BMI design. I recommend the publication of the manuscript.

One note, inspired by the request from reviewer #1 for data that is low-pass filtered in hardware – dual band data recorded with Neuropixels 1.0 might be a very useful source of new training and verification data for spike reconstruction. To be clear, however, I believe this manuscript is ready with the verification data provided.

Reviewer #1 (Remarks to the Author):

This reviewer is satisfied with all responses to questions and happy with the revised manuscript.

>> We appreciate your insightful feedback and discussion to help us improve our manuscript.

Reviewer #2 (Remarks to the Author):

The authors have largely addressed my prior comments. They could not address comment 1, but I predicted it to be a difficult task. I would like to recommend publication.

>> We appreciate your insightful feedback and discussion to help us improve our manuscript.

Reviewer #3 (Remarks to the Author):

Final review after revisions:

I believe the authors have addressed both my comments and those of the other two reviewers. The result is definitely of significant interest—reading the questions of the other reviewers gave me some more insight into the importance of such techniques in BMI design. I recommend the publication of the manuscript.

One note, inspired by the request from reviewer #1 for data that is low-pass filtered in hardware – dual band data recorded with Neuropixels 1.0 might be a very useful source of new training and verification data for spike reconstruction. To be clear, however, I believe this manuscript is ready with the verification data provided.

>> Thank you very much for your positive comments and suggestions. In follow-up research, we would work on training and evaluating our model for spike reconstruction using another dataset, such as data recorded with Neuropixels.
